# Bioactivity Deep Learning for Complex Structure-Free Compound-Protein Interaction Prediction

**DOI:** 10.1021/acs.jcim.5c00741

**Published:** 2025-09-16

**Authors:** Yaowen Gu, Song Xia, Qi Ouyang, Yingkai Zhang

**Affiliations:** † Department of Chemistry, 5894 New York University, New York, New York 10003, United States; ‡ Simons Center for Computational Physical Chemistry at New York University, New York, New York 10003, United States; § NYU-ECNU Center for Computational Chemistry at NYU Shanghai, Shanghai 200062, China

## Abstract

Protein–ligand binding affinity assessment plays a pivotal role in virtual drug screening, yet conventional data-driven approaches rely heavily on limited protein–ligand crystal structures. Structure-free compound-protein interaction (CPI) methods have emerged as competitive alternatives, leveraging extensive bioactivity data to serve as more robust scoring functions. However, these methods often overlook two critical challenges that affect data efficiency and modeling accuracy: the heterogeneity of bioactivity data due to differences in bioassay measurements and the presence of activity cliffs (ACs)—small chemical modifications that lead to significant changes in bioactivity, which have not been thoroughly investigated in CPI modeling. To address these challenges, we present CPI2M, a large-scale CPI benchmark data set containing approximately 2 million bioactivity data points across four activity types (*K*_i_, *K*_d_, EC_50_, and IC_50_) with AC annotations. Moreover, we developed GGAP-CPI, a complex structure-free deep learning model trained by integrated bioactivity learning and designed to mitigate the impact of ACs on CPI prediction through advanced protein representation modeling. Our comprehensive evaluation demonstrates that GGAP-CPI outperforms 12 target-specific and 7 general CPI baselines across 4 scenarios (general CPI prediction, rare protein prediction, transfer learning, and virtual screening) on 7 benchmarks (CPI2M, MoleculeACE, CASF-2016, MerckFEP, DUD-E, DEKOIS-v2, and LIT-PCBA). Furthermore, GGAP-CPI is able to not only deliver stable bioactivity predictions but also measure prediction uncertainty and enrich binding pocket residues and interactions, underscoring its applicability to real-world bioactivity assessments and virtual drug screening.

## Graphical abstract

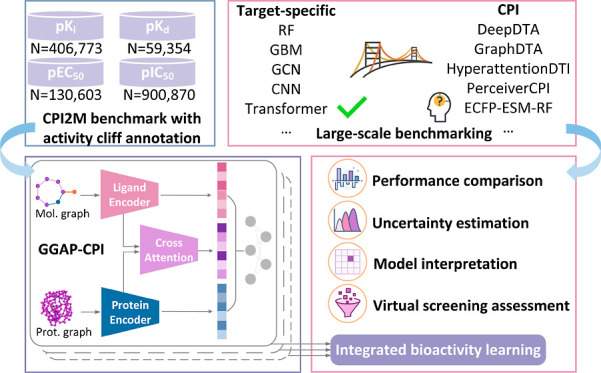

## Introduction

Accurate binding affinity prediction to given protein–ligand complexes is of the greatest importance for the scoring function (SF) in the structure-based virtual screening (SBVS) method. The recent progress of machine learning (ML) has enabled great advancements in ML-based scoring function development.^1–5^ Such data-driven binding affinity prediction approaches heavily rely on data scale and the quality of complex crystal structures,^6,7^ limiting their performance and applicability since the current crystal structure databases contain insufficient chemical and protein structure spaces (e.g., 22,920 protein–ligand complexes in PDBbind 2024 ^8^). A more general field, compound-protein interaction (CPI) prediction, has gradually gained researchers’ attention since it focuses on directly predicting binding affinities or bioactivities for protein–ligand pairs without complex structures required. Such structure-free CPI methods place far fewer constraints on both training data and inference inputs than ML-based scoring functions with substantially larger-scale CPI data available for model training (e.g., ∼1.6 M assays in ChEMBL^9^ and 1.1 M binding measurements in BindingDB^10^). Previous studies have also advocated training bioactivity prediction models on more diverse data sets, rather than relying exclusively on potentially biased crystal-structure collections such as PDBBind.^11^

CPI modeling has rapidly advanced in recent years, with some pioneering approaches outperforming traditional docking and scoring functions such as AutoDock Vina,^12^ Gnina,^13^ RF-Score,^14^ and Δ_Vina_RF^15^ in both affinity prediction accuracy and virtual screening enrichment.^16,17^ For instance, ActFound constructs a target-specific foundation model from 2.3 million bioactivity data points (ChEMBL, BindingDB), delivering robust, assay-agnostic bioactivity predictions;^18^ MBP’s multitask, structure-based pretraining on large-scale docking complexes yields transferable binding representations;^19^ DTIGN employs semisupervised learning on docking poses and crystal structures to jointly capture binding modes and activities with attention mechanisms;^20^ and BALM uses parameter-efficient fine-tuning of protein and ligand language models to learn highly detailed structure–activity relationships even under challenging data splits.^21^ The consistent advantage of these approaches stems from their access to vast, diverse bioactivity data sets, which enable the models to learn nuanced structure–activity patterns. However, the availability of a vast amount of bioactivity data for training CPI models presents both opportunities and challenges.

From the data heterogeneity aspect, a critical issue is the comparability and uncertainty of bioassay data with different activity types, which can potentially limit the effectiveness of data-driven CPI methods in practical virtual screening. For instance, IC_50_ values are influenced by the reaction types, concentrations of enzymes, inhibitors, substrates, and other experimental conditions, while *K*_i_ values are intrinsic thermodynamic quantities that depend solely on the interactions between enzymes and inhibitors.^22^ It is generally accepted that thermodynamic affinity properties, such as *K*_i_ and *K*_d_, offer the highest quality and consistency. In contrast, other bioactivity measures like EC_50_, IC_50_, and percentage inhibition tend to be less reliable.^23^ Given these inconsistencies, directly converting all bioactivity measurements to a single, unified type is impractical. However, as modern ML models have been developed to readily capture linear relationships, the highly correlated bioactivity data across different assay types can still be leveraged together to train a robust bioactivity predictor, therefore enabling effective integration of such cross-type signals.^24^ However, blindly combining bioactivity data from different assays could introduce significant noise, thereby impairing the performance of data-driven CPI models.^25^ A recent study has also demonstrated that careful data integration and multistage machine learning modeling can successfully leverage multiple data types to improve kinase bioactivity prediction.^26^ These pieces of evidence highlight the need for comprehensive data processing and tailored CPI modeling strategies to effectively utilize large-scale, but often noisy, bioactivity data.

From the data fitness aspect, one critical assay assessment phenomenon—activity cliff (AC)—could significantly influence the affinity/bioactivity prediction performances. AC is defined as pairs of structurally similar compounds but having significant differences in bioactivities, which represents the discontinuity in activity landscapes in structure–activity relationships.^27,28^ For ML-based data-driven approaches that are always trained based on the “chemical similarity principle”, the AC molecules are typically recognized as abnormal samples and lead to significantly inaccurate affinity/bioactivity prediction^29^ and hinder model training.^30^ Meanwhile, the small structure changes between positive ACs and negative ACs cause a high false positive rate that is particularly harmful for virtual screening methods.^31^ Previous AC studies have explored and emphasized the large performance differences between ACs and non-ACs for target-specific bioactivity prediction models,^29,31–34^ while some studies applied the AC concept for binding affinity and bioactivity prediction-related tasks such as hit-to-lead optimization^35,36^ and structural alert development.^37^

Although ACs are recognized as a major challenge for bioactivity prediction, their impact on general CPI methods remains largely unexplored. Medicinal chemistry findings suggest that CPI models may partially mitigate AC-related performance discrepancies through two complementary mechanisms. First, protein structures help to better describe ACs. The identification of ACs is thought to relate to the binding conformations.^27,28^ Therefore, compared to most previous studies that consider only ligand features,^29,31–33^ CPI methods leverage both ligand and protein representations and may thus alleviate AC-induced discrepancies—an effect that still warrants systematic investigation. Second, more diverse data include a wider landscape of ACs. The “activity ridge” has been observed across multiple targets’ bioactivity differences,^38^ and chemical structures of distinct AC pairs often correlate strongly.^39^ Integrating bioactivity data from a broad range of targets can, therefore, capture a more complete AC landscape and enhance model training. However, existing benchmarks either cover only a narrow set of targets^31^ or lack quantitative bioactivity labels,^32^ preventing a comprehensive assessment of how CPI approaches exploit protein context and data diversity to overcome AC challenges.

Inspired by these observations and challenges, we first construct a large-scale CPI data set (CPI2M) with activity cliff annotations and multiple activity types available (*K*_i_, *K*_d_, EC_50_, and IC_50_). Then, a crystal structure-free CPI model, called GGAP-CPI (protein Graph and ligand Graph network with Attention Pooling for Compound-Protein Interaction prediction, Figure 1), is proposed for accurate CPI bioactivity prediction. GGAP-CPI adopts a pretrained ligand encoder (KANO^40^) and a protein embedding generator (ESM-2 ^41^) for advanced representation learning, with a multihead cross-attention pooling assembled to simulate and aggregate the interactions between ligand atoms and protein residues. GGAP-CPI is trained with an integrated bioactivity learning regime to be flexible for general CPI bioactivity prediction.

**Figure 1:**
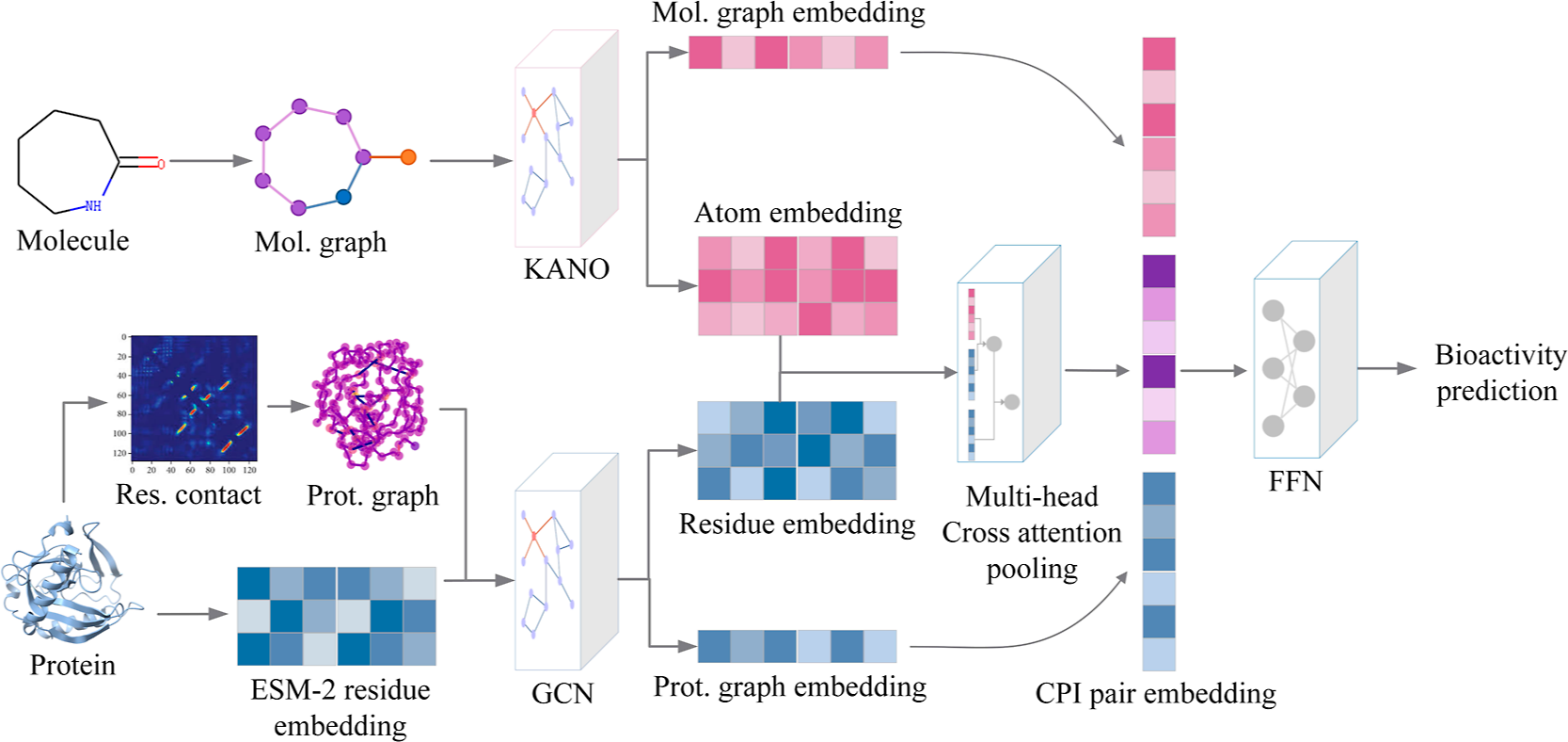
GGAP-CPI architecture. This model is composed of two encoders: the pretrained ligand encoder takes a ligand molecular graph as an input to compute a molecular graph and atom-level embedding; the protein encoder takes a protein graph and pretrained residue embedding as inputs to compute a protein graph and residue-level embedding. A multihead cross-attention pooling module is used to generate CPI complex representation based on the previous multiscale ligand and protein embeddings. Finally, a feed-forward network is constructed for bioactivity prediction.

Regarding the main results, the data set quality of CPI2M is first investigated by overlapping analysis and chemical space visualization to ensure the validity of CPI2M for overall and AC performance evaluation. Then, we conduct extensive comparison experiments on CPI2M and MoleculeACE^31^ with internal/external/transfer learning validation data set settings, highlighting the superiority of GGAP-CPI compared to various baselines on multiple general and challenging scenarios. Regarding AC samples, our results indicate that general CPI prediction—by incorporating protein information into the model—yields more accurate results than target-specific approaches. Meanwhile, considering practical virtual screening assessment, CASF-2016,^42^ MerckFEP,^43^ DUD-E,^44^ DEKOIS-v2,^45^ and LIT-PCBA^46^ benchmarks are also introduced, indicating GGAP-CPI is comparable to other widely used scoring function methods with superior ranking and scoring powers, highlighting the potential of the GGAP-CPI lead-optimization stage. In all, building on previous studies, our study promotes the current investigation of activity cliffs from target-specific exploration to integrated CPI prediction with substantially larger data sets, more comprehensive evaluations, and more effective methodology designs.

## Results and Discussion

We delved into our study by proposing and answering five questions to investigate the contribution of our proposed CPI2M benchmark data set and GGAP-CPI model, including the following:

Q1: How is the data set quality and variety of the CPI2M benchmark?—Overview of the benchmark data set.

Q2: Does GGAP-CPI outperform other competitive target-specific and CPI baselines in different scenarios and validations, considering both general metrics and AC-related metrics?—Comprehensive bioactivity prediction performance comparison.

Q3: Is every key component (e.g., ligand encoder, protein encoder, and attention pooling) in GGAP-CPI nonredundant and valuable for model performance?—Ablation study for GGAP-CPI.

Q4: Can GGAP-CPI serve effectively as a scoring function for virtual screening?—Virtual screening assessment.

Q5: Is GGAP-CPI applicable to broader applications?—Bioactivity uncertainty estimation and protein pocket enrichment.

### Overview of the Benchmark Data Set

#### Overlaps in Source Data

The CPI2M data set, derived from the integration of EquiVS and Papyrus data and originating from various public databases, necessitates a thorough examination of data overlaps to assess the need for data integration and redundancy elimination. Figure 2A illustrates the extent of overlap between EquiVS and Papyrus in terms of ligands, proteins, unique CPI (combined unique ligand and protein identifiers), and unique bioactivity profiles (combining unique identifiers for ligand, protein, and bioactivity), while Figure S1 details the overlaps across activity type-based subsets in CPI2M. The results reveal that approximately 10% of the CPI data between EquiVS and Papyrus is conflicting and requires resolution and exclusion to ensure data integrity. This level of redundancy is deemed acceptable for the purposes of integrating these data sets. Moreover, while there are moderate overlaps in ligand molecules between EquiVS and Papyrus, the overlaps in the protein data are notably larger. This suggests EquiVS and Papyrus may share similar chemical and protein structural spaces, warranting further analysis to understand the implications of these overlaps on the data set’s diversity and utility in CPI prediction and AC effect estimation.

**Figure 2:**
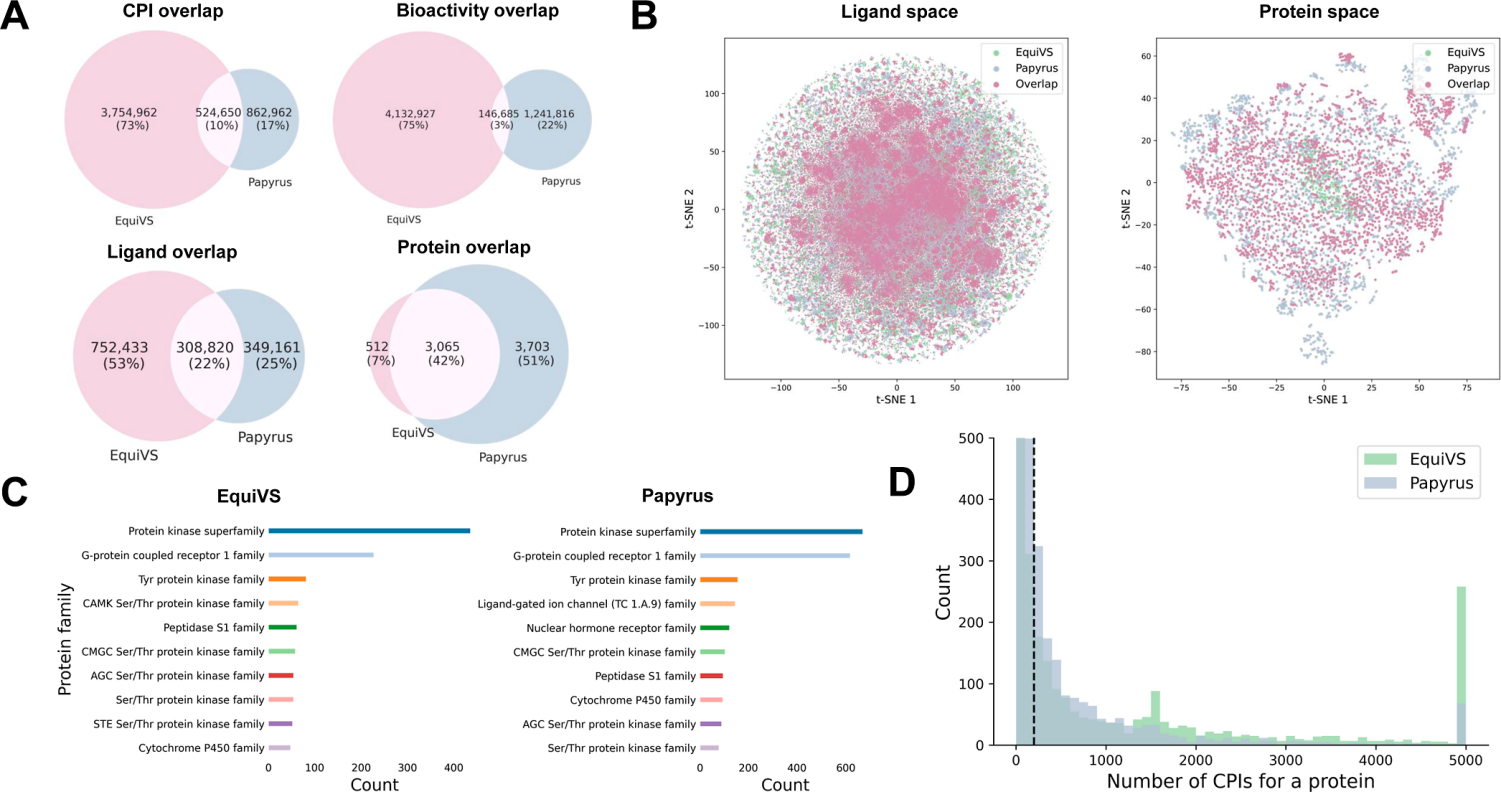
(A) Overlaps regarding EquiVS and Papyrus source data in CPI2M. (B) Ligand and protein structure space visualization by t-SNE reduced features. (C) Top 10 InterPro protein families for EquiVS and Papyrus source data in CPI2M. (D) Distribution of number of assays for target proteins.

#### Chemical and Protein Spaces

To investigate the chemical and protein spaces within the CPI2M data set, we utilized molecular Extended Connectivity Fingerprints (ECFPs) for ligand representation and Evolutionary Scale Modeling (ESM-2) pretrained embeddings for protein representation. Feature reduction and visualization were performed using t-Distributed Stochastic Neighbor Embedding (t-SNE), with the visual results displayed in Figure 2B. Additionally, PCA-based visualizations are presented in Figure S2. The visualization of ligand chemical space reveals that the molecular structures from EquiVS, Papyrus, and their intersecting data set occupy similar structural spaces. This coherence in structural adjacency suggests that the integration of these data sets is unlikely to introduce significant shifts or discontinuities within the CPI2M data set, supporting the validity of our data integration approach. In contrast, the visualization of protein space is notably sparser, featuring some isolated samples. A further investigation into the protein diversity was conducted by analyzing the top 10 protein families from the InterPro^47^ database within the EquiVS and Papyrus data sets, as depicted in Figure 2C. This result highlights a high degree of overlap (8 out of 10) among the most prevalent protein families, such as the protein kinase superfamily, G-protein-coupled receptor 1 family, and Tyr protein kinase family. This overlap signifies that the integration of EquiVS and Papyrus data sets not only complements but also significantly enriches the protein diversity in the CPI2M data set without incorporating detrimental out-of-distribution samples. The examination of unique measurements and structural spaces for the CPI2M source data validates the comprehensive nature of our data curation and processing strategies.

#### Protein Assay Distributions

We further analyzed the distribution of the number of CPIs for each target protein within the CPI2M data set to illustrate the overall sparsity of assay data. As depicted in Figure 2D, the analysis reveals that most proteins are associated with fewer than 200 available assay measurements. This low number of measurements raises challenges for accurately assessing the AC effect and complicates target-specific model training. To address these challenges, we divided the CPI2M data set into three subsets: the CPI2M-main training set, the CPI2M-main validation set, and the CPI2M-few test set. CPI2M-main is utilized for model training (CPI2M-main train) and internal validation (CPI2M-main valence) by applying an 8:2 AC-specific data splitting. It comprises targets with assay measurements exceeding the 200 threshold, providing a robust data set for general model performance evaluation. The CPI2M-few test set includes targets with fewer than 200 measurements and is designated for validating model performance on “unseen” proteins, posing a more challenging scenario. Additionally, we incorporated an external benchmark, MoleculeACE,^31^ specifically for transfer learning validation. The statistical characteristics of the CPI2M-main train, CPI2M-main val, CPI2M-few test, and MoleculeACE benchmark data sets are detailed in Table 1, providing a comprehensive overview of the bioactivity data utilized in our study.

**Table 1:** Summary of Data Sets and Their Characteristics

data set	activity type	num.	num. mol.	num. prot.	avg. bioactivity	std. bioactivity	% AC
CPI2M-main train	*K*_i_	272,085	109,185	481	6.50	1.42	25.37
	*K*_d_	3427	2627	21	6.91	1.59	34.08
	EC_50_	70,924	51,656	178	5.80	1.57	25.06
	IC_50_	599,293	359,692	1115	6.15	1.47	30.60
CPI2M-main val	*K*_i_	69,159	41,078	481	6.50	1.42	25.43
	*K*_d_	910	805	21	6.87	1.62	33.85
	EC_50_	18,008	16,218	178	5.81	1.56	25.14
	IC_50_	152,648	122,370	1115	6.15	1.47	30.55
CPI2M-few test	*K*_i_	54,939	37,612	1688	6.22	1.55	
	*K*_d_	42,646	10,404	1133	5.42	1.28	
	EC_50_	42,294	28,817	1504	6.00	1.48	
	IC_50_	123,299	90,000	3117	5.82	1.39	
MoleculeACE	*K*_i_	40,220	29,594	23	7.30	1.35	38.18
	EC_50_	8494	6625	7	6.70	1.21	40.93

### Bioactivity Prediction Performance Comparison

#### Comparison on the Internal Validation Data Set

To evaluate the superiority of GGAP-CPI in CPI binding affinity and bioactivity prediction relative to other methods, we employed four data sets for internal validation, each characterized by distinct activity types: *K*_i_, *K*_d_, EC_50_, and IC_50_. The performance was assessed using general metrics (root-mean-square error: RMSE; Pearson’s correlation coefficient: PCC; Spearman’s correlation coefficient: SRCC) and activity cliff-specific metrics (RMSE_cliff_, PCC_cliff_, and SRCC_cliff_), with results for the four data sets in CPI2M shown in Figures 3, S3, and S4 for an overall performance evaluation. Task-specific performance across all protein subsets is detailed in Tables S1–S4. These results demonstrate that GGAP-CPI consistently surpasses both target-specific and general CPI baselines across most bioactivity-specific data sets, maintaining its superiority in activity cliff-specific metrics across all data sets. Specifically, GGAP-CPI ranks first in all metrics on the CPI2M-main-*K*_i_, -*K*_d_, and -IC_50_ data sets, with notable improvements (RMSE: 7.77% on *K*_i_, 5.95% on *K*_d_, and 9.80% on IC_50_; RMSE_cliff_: 6.93% on *K*_i_, 1.72% on *K*_d_, and 3.59% on IC_50_) achieved compared to the second-best results. On EC_50_ data, GGAP-CPI ranked second in SRCC and PCC_cliff_, while it ranked third on other metrics. Notably, previously proposed CPI baselines (i.e., DeepDTA, GraphDTA, HyperattentionDTI, and PerceiverCPI) fail to outperform our simple ML-based CPI baseline (i.e., ECFP-ESM-RF), further underscoring the significant advancements of GGAP-CPI as the only DL-based CPI method to outperform the simple model. This observation aligns with previous studies^31,48^ that have noted simple ML-based models often outperform DL-based ones in molecular property and bioactivity prediction tasks. These results confirm that GGAP-CPI more accurately predicts binding affinities for compound-protein pairs compared to various baselines. Even for AC compound-protein pairs, which represent highly sensitive and challenging samples within the CPI data set, GGAP-CPI still achieves commendable accuracy and significantly improves the AC-specific performances.

**Figure 3:**
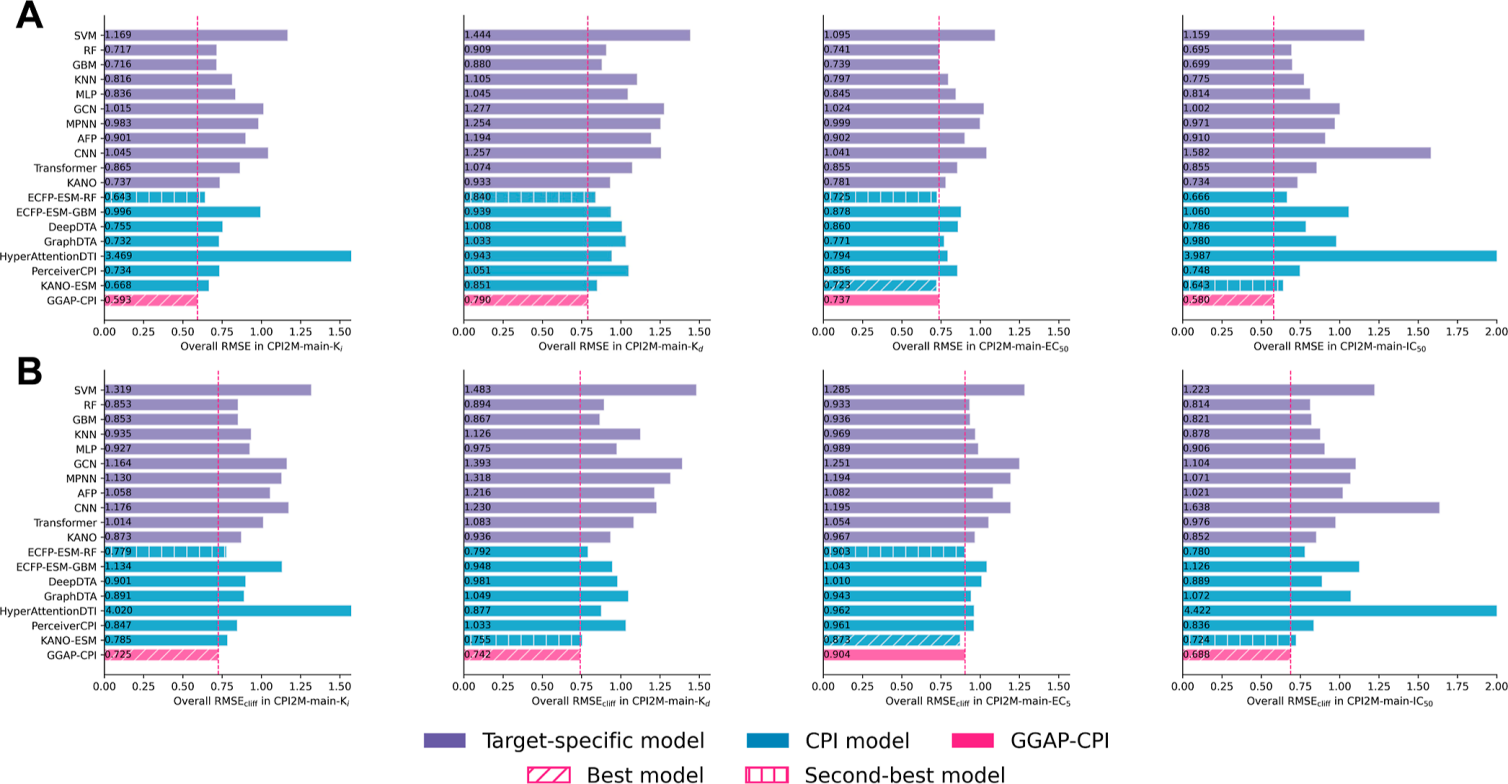
Comparison of overall general root-mean-square error (RMSE) and AC-specific RMSE (RMSE_cliff_) for GGAP-CPI (rose red), target-specific baselines (purple), and CPI baselines (steel blue) on the internal CPI2M-main validation sets (*K*_i_, *K*_d_, EC_50_, IC_50_). Diagonal hatching denotes the best-performing model, and vertical-line hatching denotes the second-best. Lower values indicate better performance. (A) Overall RMSE; (B) overall RMSE_cliff_. The RMSE_cliff_ is computed by calculating RMSE for AC data in the CPI2M-main validation set.

Further comparison with target-specific methods (e.g., RF) and CPI methods (e.g., ECFP-ESM-RF) highlights the effectiveness of well-designed CPI approaches. These outperform target-specific methods significantly, suggesting that integrating protein information and considering multiple target subsets could enhance the binding affinity and bioactivity prediction accuracies. Our internal validation results offer a comprehensive overview of performance across different affinity/bioactivity types, data scales, and included targets, consistently supporting the superior performance of GGAP-CPI, especially with AC molecules.

We then compared predictive errors on AC versus non-AC compounds. On the CPI2M-main *K*_i_, EC_50_, and IC_50_ data sets, AC compounds consistently exhibited larger errors (RMSE_cliff_ > RMSE) than non-AC compounds, highlighting the difficulty that abnormal bioactivity differences pose for CPI models. By contrast, the CPI2M-main-*K*_d_ data set displayed the opposite pattern across GGAP-CPI and most baselines. We attribute this discrepancy to the relatively small size and limited diversity of the *K*_d_ data set, which can distort performance estimates when sample counts are low.

#### Comparison on the External Validation Data Set

Following the analysis of internal validation results, we extended method evaluation to include GGAP-CPI’s performance on unknown proteins and rare samples. We compiled an external validation (CPI2M-few test sets) consisting of proteins with fewer than 200 available assay data points. This data set is designed to simulate the challenges of practical zero-shot virtual screening scenarios, where target-specific methods are inapplicable due to the absence of reference data for model training.

We assessed the performance of GGAP-CPI against other CPI baseline methods across a few CPI2M test sets. The results, presented in Figures 4 and S5 (AC-specific performances) and Tables S5–S8 (target-specific performances), reveal that all CPI methods experienced significant performance declines in this demanding experimental context. Many baselines were incapable of generating valid predictions with their Pearson’s correlation coefficient (PCC) scores falling below 0.5. In contrast, GGAP-CPI demonstrated the best performances on nearly all metrics and data sets, showcasing moderate predictive reliability. Especially on PCC, the relative improvements achieved by GGAP-CPI compared to the second-best results are 23.45%, 18.67%, 78.66%, and 15.09% on *K*_i_, *K*_d_, EC_50_, and IC_50_ data, respectively. These findings underscore the robustness and broader applicability of GGAP-CPI in practical CPI bioactivity prediction scenarios, particularly in settings that challenge conventional target-specific approaches.

**Figure 4:**
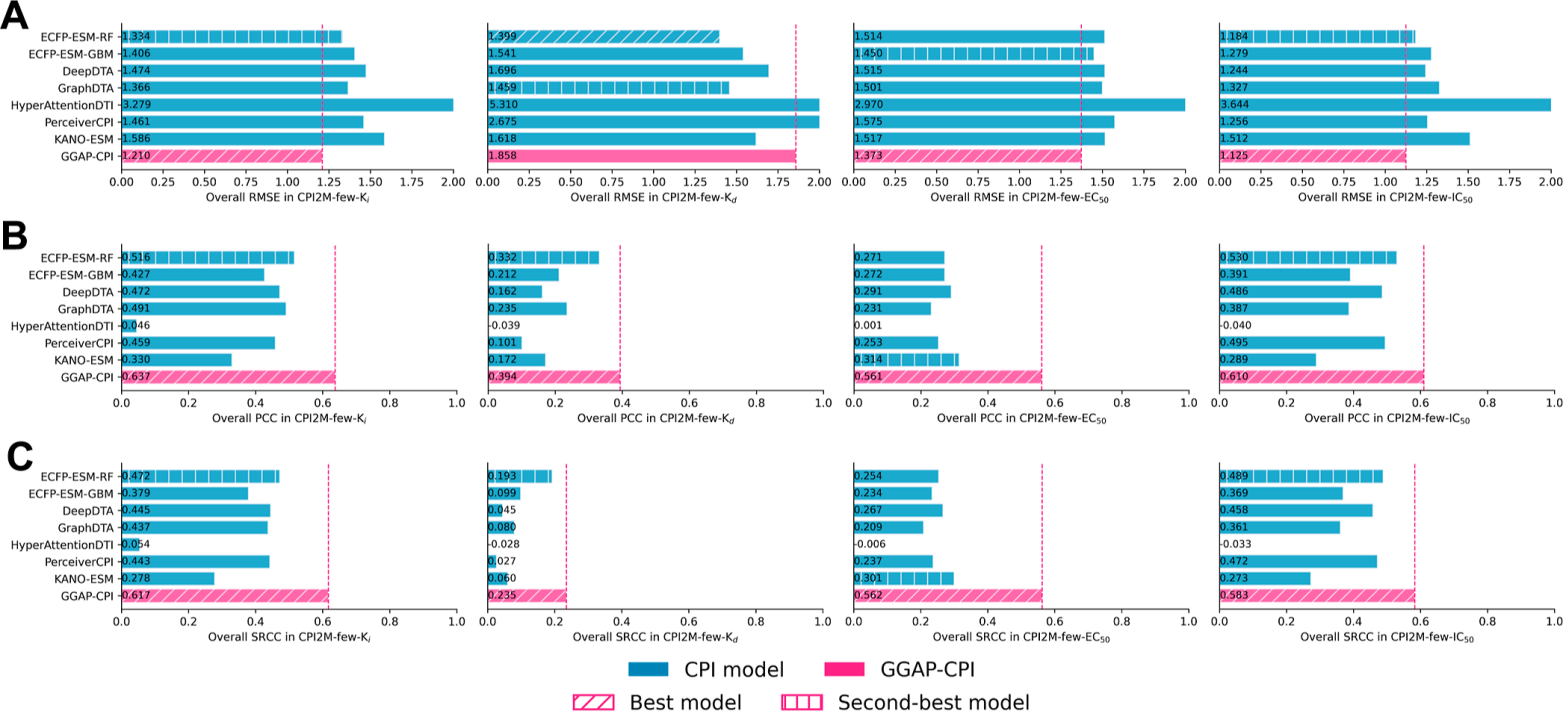
Comparison of overall general performance metrics—root-mean-square error (RMSE), Pearson’s correlation coefficient (PCC), and Spearman’s rank correlation coefficient (SRCC)—for GGAP-CPI (rose red) and CPI baselines (steel blue) on the CPI2M-few test sets (*K*_i_, *K*_d_, EC_50_, IC_50_). Diagonal hatching marks the best-performing model, and vertical-line hatching marks the second-best. Lower RMSE and higher PCC/SRCC values indicate better performance. (A) Overall RMSE; (B) overall PCC; (C) overall SRCC.

#### Comparison on the Transfer Learning Validation Data Set

In our study, we employed a widely used activity cliff estimation benchmark, MoleculeACE, as the validation data set for transfer learning. This is to simulate downstream tasks in which ample target-specific bioactivity data are available to refine further general CPI models. We fine-tuned GGAP-CPI on MoleculeACE-*K*_i_ and -EC_50_ data sets, which is noted as “GGAP-CPI-ft”. To ensure the integrity of the training process, any overlapping data in CPI2M were excluded to prevent data leakage. We evaluated the performance of GGAP-CPI-ft, other target-specific, and CPI baselines on MoleculeACE-*K*_i_ and MoleculeACE-EC_50_ data sets. As shown in Figure 5, with target-specific average results shown in Tables S9 and S10, GGAP-CPI-ft significantly outperformed other baselines on all data sets, with notable relative improvements achieved compared to suboptimal baseline results (e.g., *K*_i_: 8.27% for RMSE and 3.20% for RMSE_cliff_; EC_50_: 12.02% for RMSE and 10.83% for RMSE_cliff_).

**Figure 5:**
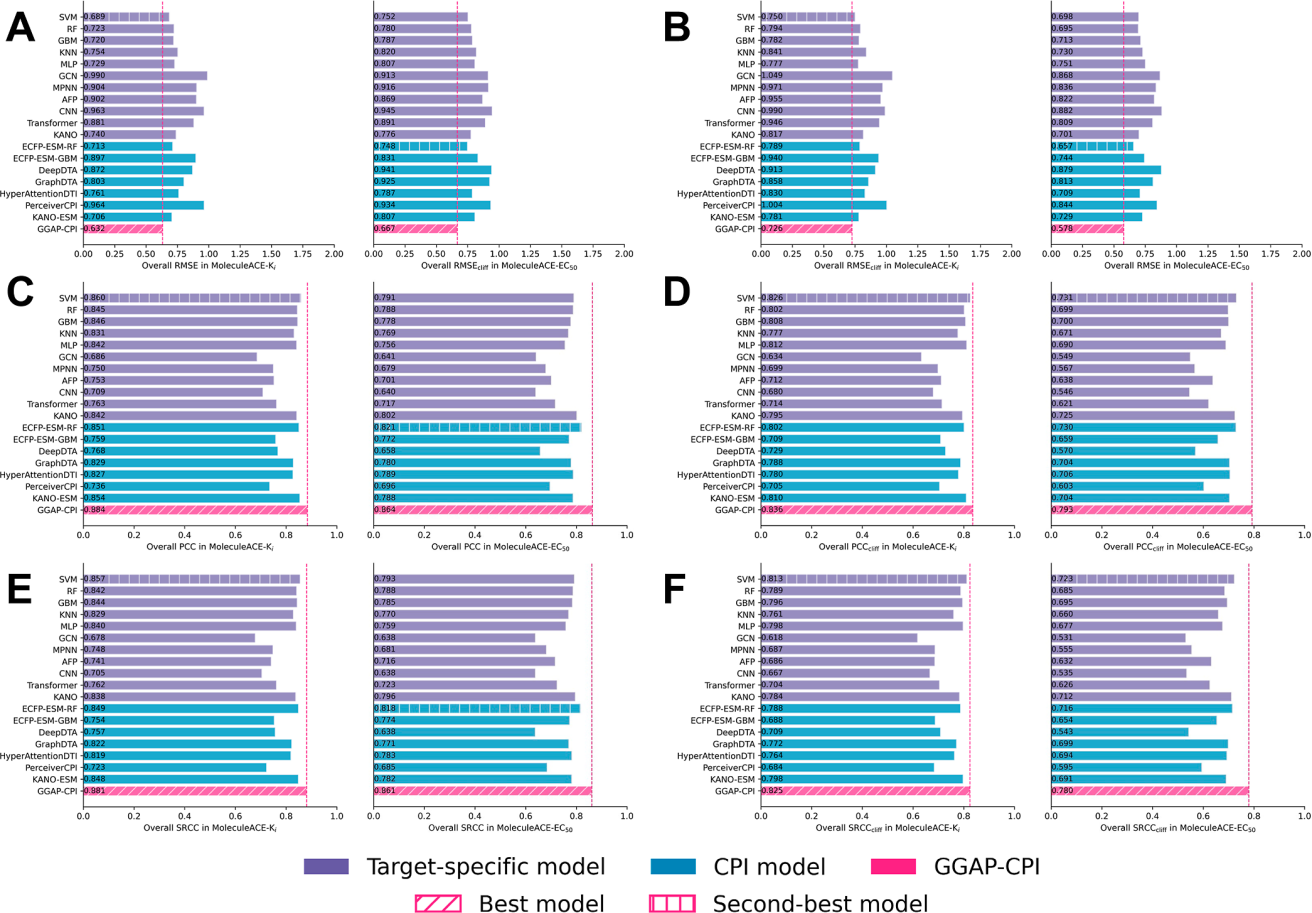
Comparison of overall general performance metrics—root-mean-square error (RMSE), Pearson’s correlation coefficient (PCC), and Spearman’s rank correlation coefficient (SRCC)—and AC-specific metrics (RMSE_cliff_, PCC_cliff_, SRCC_cliff_) for GGAP-CPI (rose red), target-specific baselines (purple), and CPI baselines (steel blue) on the MoleculeACE transfer learning validation sets (*K*_i_, EC_50_). Diagonal hatching identifies the best-performing model, and vertical-line hatching identifies the second-best. Lower RMSE and higher PCC/SRCC values indicate superior performance. (A) Overall RMSE; (B) overall RMSE_cliff_; (C) overall PCC; (D) overall PCC_cliff_; (E) overall SRCC; (F) overall SRCC_cliff_.

### Ablation Study for GGAP-CPI

We conducted an ablation study to empirically assess the individual contributions of various components within GGAP-CPI to model performance, which compared the full GGAP-CPI model with five variants, each lacking one core module:

KANO: The KANO model can be regarded as a GGAP-CPI variant with only a ligand encoder. The protein encoder and multihead cross-attention mechanism are removed.

GGAP-CPI-w/o Mol. Encod.: This variant operates without the KANO ligand encoder, utilizing an ECFP molecule fingerprint instead. This setup helps assess the impact of the pretrained ligand encoder integrated within GGAP-CPI.

GGAP-CPI-w/o Prot. Encod.: In this configuration, the graph convolutional network (GCN) protein encoder is replaced by a simple linear layer, evaluating the role of the protein graph construction and graph convolutional feature updating.

GGAP-CPI-w/o ESM-2 Emb.: This model excludes the ESM-2 residue embedding, substituting it with a residue type of one-hot embedding. This change highlights the significance of protein embedding derived from the pretrained protein language model.

GGAP-CPI-w/o CroAtt. Pool.: This variant lacks the multihead cross-attention mechanism, instead combining protein and ligand embeddings through simple concatenation. This tests the effectiveness of the attention-based pooling mechanism.

GGAP-CPI-w/o Int. Ensemb.: This variant retains the full GGAP-CPI architecture but removes integrated bioactivity learning across the activity types. Instead, the training data are split into four separate subsets by activity type (*K*_i_, *K*_d_, EC_50_, and IC_50_), and the model is trained independently on each subset to predict bioactivity for that specific measurement.

As depicted in Figure 6, the results demonstrate that GGAP-CPI outperformed all variants on three activity-type data points (*K*_i_, *K*_d_, and IC_50_), affirming the synergy and effectiveness of its comprehensive design. Notably, KANO and the variants without the molecular encoder and the ESM-2 embeddings—GGAP-CPI-w/o Mol. Encod. and GGAP-CPI-w/o ESM-2 Emb.—showed the worst performance across all data sets, underscoring the critical contributions of the pretrained ligand encoder and protein embeddings to model accuracy. Although the other architectural components, such as protein graph convolution and multihead cross-attention pooling, also enhanced performance, their impact was comparatively moderate. GGAP-CPI’s performance on EC_50_ data was somewhat lower, likely because the predominance of IC_50_ data points in the training set skewed the label distribution and introduced a minor bias. Nevertheless, our integrated bioactivity training nevertheless yielded significantly better zero-shot EC_50_ predictions, as demonstrated in Figure 3. These findings from the ablation study suggest that each component of GGAP-CPI is essential and effectively contributes to its performance, with no redundancy in the model’s architecture.

**Figure 6:**
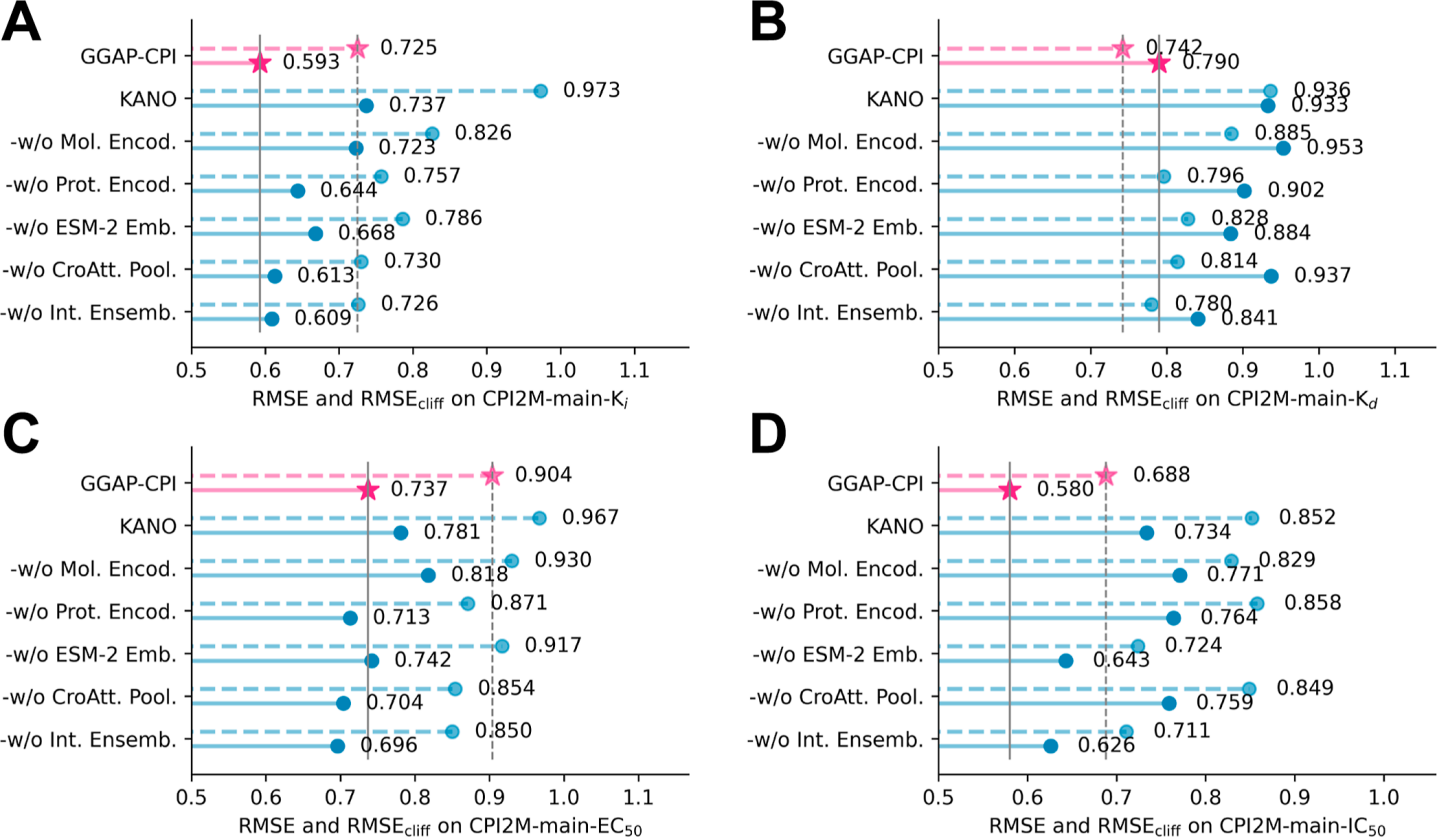
Comparison of overall and AC-specific performance, measured by RMSE (solid line) and RMSE_cliff_ (dotted line), for GGAP-CPI and its variants across the four CPI2M-main internal validation sets. Lower values indicate better performance. (A) Performances on CPI2M-main-*K*_i_, (B) performances on CPI2M-main-*K*_d_, (C) performances on CPI2M-main-EC_50_, and (D) performances on CPI2M-main-IC_50_.

### Virtual Screening Assessment

Protein–ligand binding affinity prediction methods typically serve as scoring functions (SFs) for structure-based virtual screening. To evaluate the applicability of GGAP-CPI and other CPI baselines as SFs, we conducted a comprehensive virtual screening assessment using five well-recognized benchmarks: CASF-2016,^42^ MerckFEP,^43^ DUD-E,^44^ DEKOIS-v2,^45^ and LIT-PCBA.^46^ Among these benchmarks, CASF-2016 and MerckFEP are used to evaluate ranking and scoring power, while LIT-PCBA, DUD-E, and DEKOIS-v2 are used to assess the screening power. We compared GGAP-CPI to multiple existing SFs. A brief introduction of these compared SFs is available in Supporting Information.

#### Assessment on Scoring and Ranking Power

Scoring power describes the capacity of a SF to generate binding scores that exhibit a linear correlation with experimental binding data.^42^ Meanwhile, ranking power refers to the ability of a SF to correctly rank the known ligands of a certain target protein by their binding affinities.^42^ We highlight these two evaluation metrics as the most important ones because they directly align with the primary goal of CPI bioactivity prediction models—to accurately predict bioactivities that closely match experimental measurements. According to the original definition,^46^ the Pearson’s correlation coefficient and the Spearman’s correlation coefficient are employed to evaluate scoring power and ranking power, respectively.

For CASF-2016, we collected 19,443 protein–ligand binding complexes along with their experimental binding affinities from the PDBbind-v2020 general set,^49^ with 285 of them identified as the CASF-2016 benchmark. The ligand and protein AlphaFold 2-predicted structures and sequences were taken as input for GGAP-CPI and other CPI baselines to directly predict binding affinities. The performance results, shown in Figure 7A,B and Table S13, indicate that GGAP-CPI consistently performed more competitively than other CPI-SFs in terms of scoring power and ranking power, with relative improvements of 24.44% for ranking power compared to the next best methods (ECFP-ESM-GBM). Compared to other SFs, GGAP-CPI is comparable to traditional SFs and DL-based SFs such as AutoDock Vina,^12^ CarsiDock,^50^ and RTMScore.^51^ We also found that GGAP-CPI can be further largely improved after fine-tuning on the PDBbind-v2020 general set, achieving a scoring power of 0.857 and a ranking power of 0.818 (Table S13).

**Figure 7:**
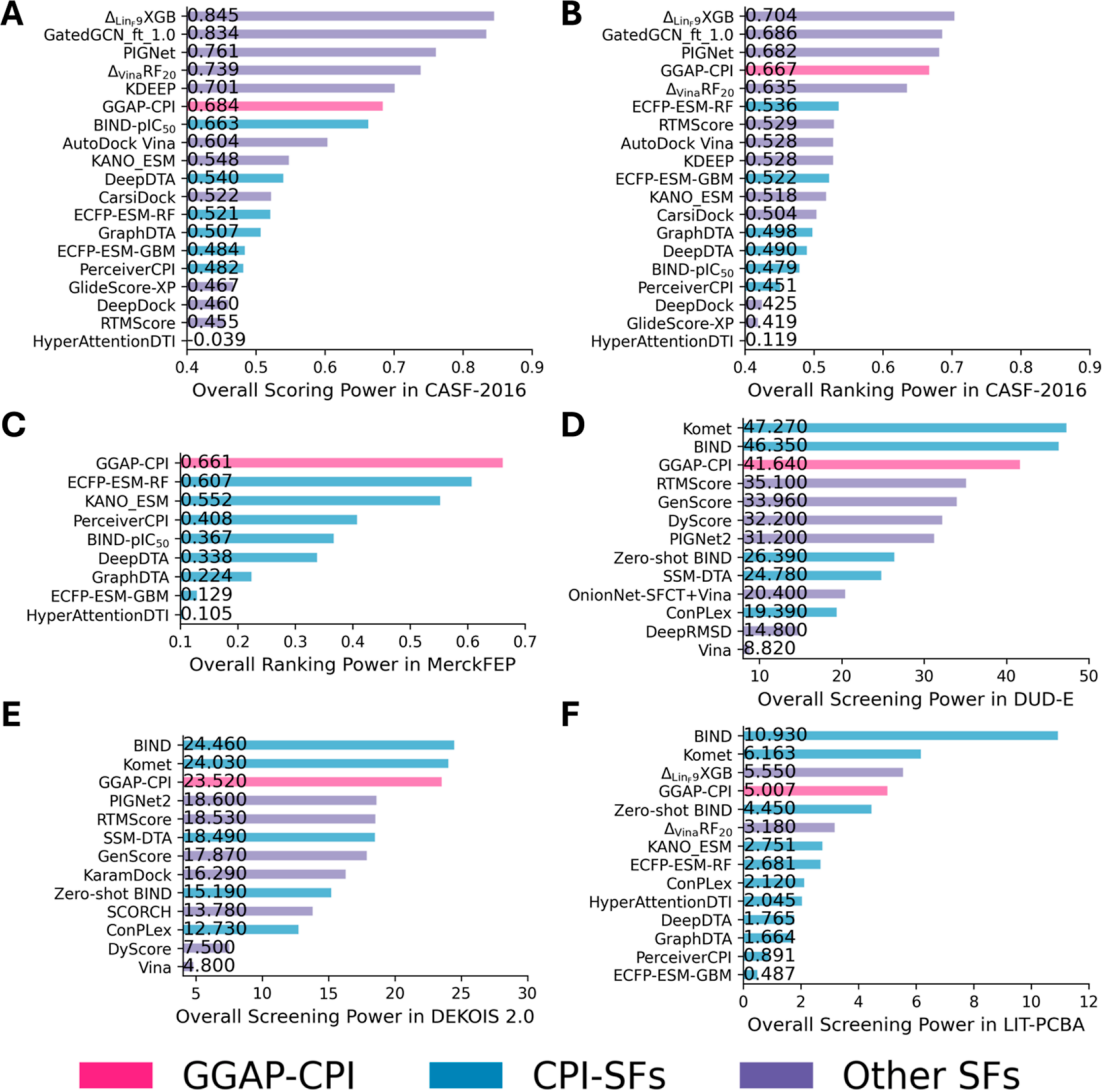
Comparison of scoring (PCC), ranking (SRCC), and screening (EF_1%_) powers for GGAP-CPI, CPI baselines, and other existing SFs. Results except for CPI-SFs are collected from refs 16, 50, 52, and 53 . CPI-SFs, except for BIND-pIC_50_,^16^ ConPLex,^54^ and Komet,^55^ are trained on CPI2M-main-IC_50_. ConPLex and BIND-pIC_50_ were collected from the original studies for assessment. Komet was trained on LCIdb following the original study. (A) Scoring power on CASF-2016. (B) Ranking power on CASF-2016. (C) Scoring power on MerckFEP. CPI-SFs, except for BIND-pIC_50_, are trained on CPI2M-main-IC_50_. (B) Ranking power on CASF-2016. (D) Screening power on DUD-E. (E) Screening power on DEKOIS-v2. (F) Screening power on LIT-PCBA.

The MerckFEP benchmark is a public virtual screening benchmark composed of 264 compounds targeting eight pharmaceutically relevant proteins with experimentally validated bioactivities. It has been widely used to evaluate the ranking power of various SFs by measuring correlations between predicted activity scores and true affinities. Since 70 data points in the Merck FEP overlap with the CPI2M-main training data, we focused our comparison on the remaining 194 data points to ensure a fair evaluation. As shown in Figure 7C and Table S14, GGAP-CPI achieved the best performance across all methods, with a relative average improvement of 8.90% over the suboptimal approach (ECFP-ESM-RF). Considering targets with fewer than five overlapping data points (Eg5, HIF-2α, PFKFB3, and SHP-2), GGAP-CPI still maintained competitive performance compared to reported results in previous studies.^56,57^

This assessment on CASF-2016 and MerckFEP demonstrates the ability of GGAP-CPI for practical virtual screening, especially for the power of accurately predicting the specific affinities and ranking binders with the highest efficacies. This would highlight GGAP-CPI’s potential in the hit optimization task, which focuses on differentiating the strongest binders among a set of structurally similar ligands.

#### Assessment on Screening Power

Screening power refers to the ability of a SF to accurately distinguish true binders from a pool of random molecules for a given target protein.^46^ A high-performing SF is typically trained on both known ligands and randomly selected decoys to better simulate the actual positive-to-negative ratio encountered in virtual screening. In this context, the enrichment factor (EF) calculated among the top 1% of ranked ligands across all data sets is commonly used as the evaluation metric.

DUD-E is an enhanced benchmark comprising 102 diverse targets and 22,886 clustered ligands from ChEMBL, each supplemented with 50 decoys from ZINC. As shown in Figure 7D, our GGAP-CPI achieved an EF_1%_ of 41.642—slightly lower than BIND’s^16^ 46.35 and Komet’s^55^ 47.27 but outperforming most traditional and DL-based scoring functions such as Vina^12^ and RTMScore.^51^ In addition, the DEKOIS-v2 benchmark, which features 81 targets, 3239 active compounds, and approximately 97,000 decoys, further demonstrated the strength of our approach: GGAP-CPI achieved an EF_1%_ of 23.52, ranking in the top 3 among the selected baselines (Figure 7E). For LIT-PCBA, an unbiased bioactivity classification data set containing 15 targets with 8020 actives and 2,675,399 inactive molecules (a positive-to-negative ratio close to 1:1000), our method displayed comparable performance relative to other CPI-based scoring functions (Figure 7F). When compared with widely used scoring functions that incorporate docking procedures, GGAP-CPI not only achieved comparable screening power but also offers the distinct advantage of being completely independent of preprocessed docking or crystal structures, thereby delivering enhanced speed and flexibility for large-scale virtual screening.

It is worth noting that the overall screening performance of GGAP-CPI is lower than that of BIND and Komet on three benchmarks. This outcome stems from our intentional exclusion of generated decoys during model training—a strategy adopted to prevent the inclusion of misleading false negative samples that could undermine precise prediction and risk data leakage. As a result, binder/nonbinder classification models such as BIND tend to achieve competitive screening power but perform weakly on ranking and scoring, while our model excels in accurately predicting the affinities of true binders and maintains comparable ability to differentiate binders from nonbinders.

Meanwhile, since our primary goal is to propose a powerful bioactivity prediction model, we controlled for data overlap between CPI2M and the virtual screening benchmarks by removing common bioactivity data (i.e., those with the same ligand, protein, and bioactivity value) from the CPI2M training set to avoid “hard overlap”. We acknowledge that “soft overlaps”—such as those due to protein similarities, ligand similarities, and binarized bioactivity labels—may still exist. However, large-scale data sets inevitably include similar examples.^58^ For instance, PDBBind has been shown to share a substantial proportion of its complexes with similar protein structures to CASF-2016.^16,59^ Excluding all such similar data from training risks underestimating model performance and impairs real-world applicability for ML-SFs.^58^ Accordingly, we frame our results as demonstrating comparability—rather than outright superiority—and highlight GGAP-CPI’s capacity to learn and exploit similarity patterns present in its training data. Achieving perfectly fair model comparisons still remains challenging, owing to differences in similarity-cutoff criteria, training protocols, and evaluation pipelines across prior studies.

In summary, the evaluation results on CASF-2016, MerckFEP, DUD-E, DEKOIS-v2, and LIT-PCBA underscore the effectiveness of GGAP-CPI in handling unseen and imbalanced data sets. The integration of bioactivity learning, which mitigates AC-induced discrepancies, combined with a complex structure-free framework, significantly improves the flexibility, efficiency, and accuracy of virtual screening—making our approach a versatile and rapid tool for high-throughput drug discovery applications.

### Bioactivity Uncertainty Estimation and Protein Pocket Enrichment

After we comprehensively estimate the efficacy of GGAP-CPI in CPI bioactivity prediction and virtual screening, we further investigate the effect of GGAP-CPI on broader applications. Uncertainty estimation can quantify prediction confidence for bioactivity prediction models and thus benefit confident prediction screening for both positive and negative ligands. As GGAP-CPI is included with ten ensembled base models, following previous studies,^60,61^ we employed standard deviations of predictions from GGAP-CPI as the model uncertainties and explored the ability of enriching highly accurate predictions. Figure 8A shows the stable improvements of the mean absolute error (MAE) on the CPI2M-main when taking different uncertainty values as thresholds for prediction screening. The lower (stricter) uncertainty was assigned as the threshold; the lower (better) MAE was achieved on the screened subset.

**Figure 8:**
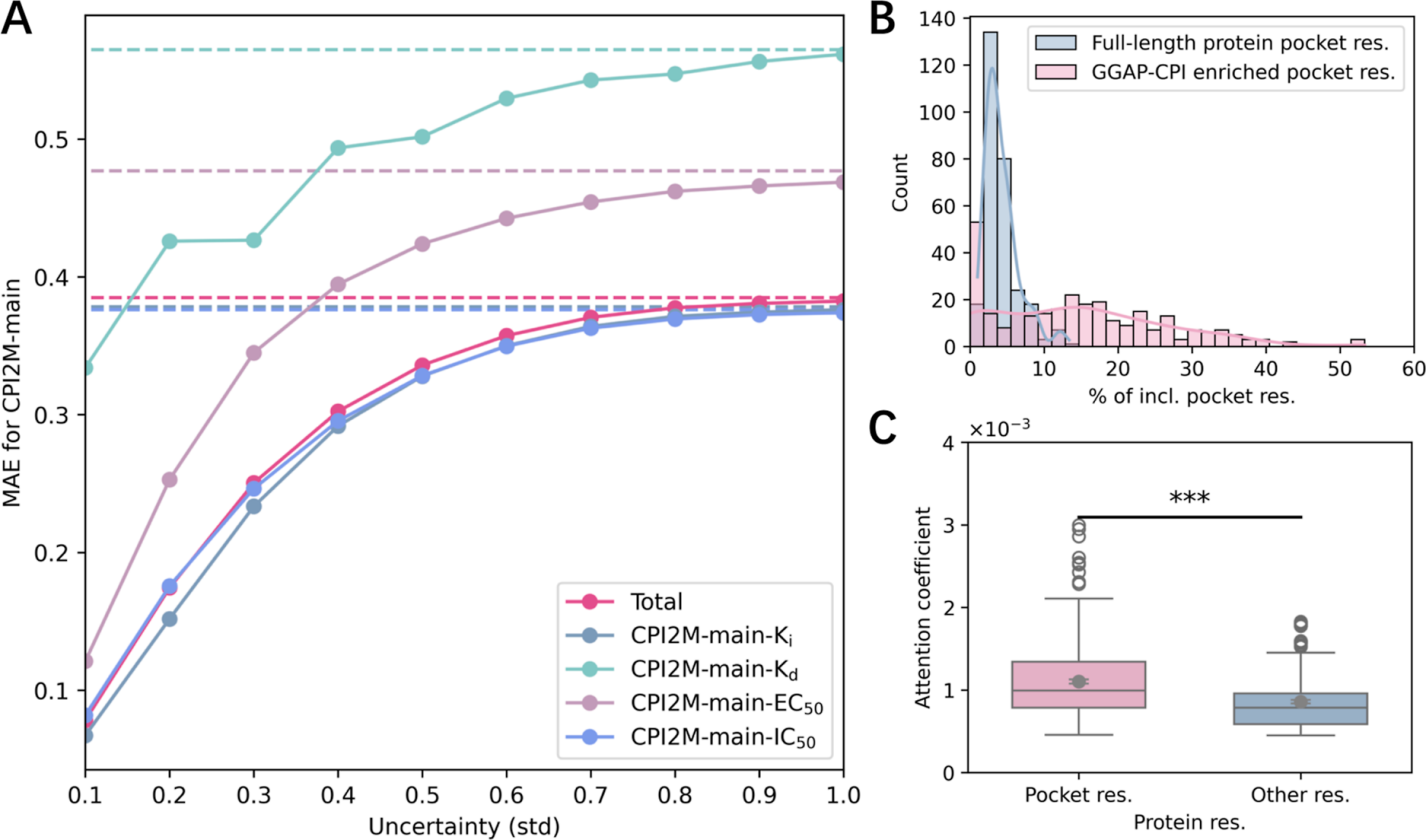
Uncertainty analysis on the CPI2M-main internal validation data set for GGAP-CPI. (A) Subset MAE vs uncertainty threshold for GGAP-CPI on the CPI2M-main internal validation sets. The corresponding uncertainty is served as the threshold to screen samples with lower uncertainty, and then MAE is calculated on these subset data. (B) Distribution of the percentages of pocket residues among full-length proteins (blue bar) and distribution of GGAP-CPI-enriched Top *N* residues by cross-attention matrix (pink bar) in the CASF-2016 data set. The protein pocket residues are identified and collected from CASF-2016 pocket PDB files. *N* equals the number of pocket residues for each protein. (C) The attention coefficient distributions of protein pocket residues and nonpocket residues in the CASF-2016 data set.

Meanwhile, GGAP-CPI incorporates a multihead cross-attention pooling module that not only simulates CPIs to generate advanced complex representations but also enhances model interpretability. By visualizing the cross-attention matrix in GGAP-CPI, we can examine potential structural binding mechanisms, highlighting key protein pocket residues with only the protein sequence information available. For each protein–ligand pair in CASF-2016, we extracted the cross-attention matrix and assigned attention coefficients to the corresponding protein residues. The distribution of Top *N* enriched residues (*N* = the number of pocket residues for a given protein) for proteins in CASF-2016 is as shown in Figure 8B. The results indicate that GGAP-CPI can effectively retrieve notable proportions of pocket residues compared with the overall distribution. Specifically, the enrichment factor for retrieving pocket residues was found to be 4.389 ± 4.100. Furthermore, there was a significant difference (*P* = 4.617 × 10^–12^, *t*-test) between the average attention coefficients for pocket residues and other residues (Figure 8C). These results showcase the potential of GGAP-CPI to provide structural insights that explain the bioactivity prediction results with an unsupervised pattern.

## Conclusion

In this study, we focus on investigating integrated bioactivity learning and performance discrepancies induced by ACs in the field of CPI prediction. A large-scale CPI benchmark with AC annotations, named CPI2M, is proposed and substantially expands the current scope of AC benchmarks in terms of data scale, enabling a comprehensive comparison across both target-specific and general CPI prediction methods. Moreover, we developed a new deep-learning-based CPI method, GGAP-CPI, which enhances CPI prediction performance by integrating bioactivity learning and mitigating the effects of ACs. GGAP-CPI consistently outperforms other methods in both general and AC-specific metrics, leveraging advanced features such as pretrained ligand encoders, protein residue embeddings, and multihead cross-attention pooling. This method maintains its superiority across various testing scenarios, including regular CPI tasks, “unseen” proteins with rare samples, and transfer learning scenarios. Results on CASF-2016, MerckFEP, DUD-E, DEKOIS-v2, and LIT-PCBA benchmarks also emphasize the competitive ranking and scoring power of GGAP-CPI in virtual screening. Further analyses highlight GGAP-CPI’s ability to quantize prediction uncertainty and offer structural insights through attention visualization. Our recent study also demonstrates GGAP-CPI’s ability to predict target-related toxicity, which helped our team achieve a fifth ranking in the Tox24 challenge.^62^

Despite these advances, significant limitations remain. Our data set curation workflow, largely inspired by the MoleculeACE study, primarily focuses on identifying potential ACs rather than specific AC molecule pairs. This approach may under-represent the nuanced effects of ACs. In future work, we plan to refine the CPI2M benchmark by incorporating pairwise AC annotations, thereby enhancing the granularity of the AC effect estimation. Meanwhile, our study demonstrates that simply transferring a target-specific task to a CPI task can significantly mitigate AC-induced performance discrepancies. However, more sophisticated designs in both the model architecture and training strategies should be considered to more directly and effectively address the AC issue, and the model interpretability for differentiating key substructures of AC molecules should be considered.

With these ongoing developments, we anticipate that both the CPI2M benchmark and the GGAP-CPI model will become increasingly suitable for practical, complex, structure-free virtual screening, ensuring more reliable and effective applications in the early stages of drug discovery.

## Materials and Methods

### Benchmark Data Set Preparation

#### Bioactivity Data Collection

Approximately 70 million bioactivity data points were aggregated from two primary sources: EquiVS^4^ and Papyrus.^63^ These sources have processed and integrated bioactivity data from several public databases, including ChEMBL,^9^ BindingDB,^10^ PubChem,^64^ Probe&Drugs,^65^ IUPHAR/BPS,^66^ EXCAPE,^67^ and literature data sets.^68^ The data available from EquiVS and Papyrus differ in terms of accessible descriptions and columns. Specifically, in EquiVS, the bioactivity data include the following: (a) molecule ID, corresponding to the source database; (b) Molecular Simplified Molecular Input Line Entry Specification (SMILES); (c) target ID, as denoted by the HUGO Gene Nomenclature Committee (HGNC); (d) bioactivity type (e.g., *K*_i_, *K*_d_, EC_50_, IC_50_); (e) bioassay type; (f) bioactivity unit; and (g) bioactivity value. Similarly, the bioactivity data in Papyrus are outlined as (a) source ID, which links back to the initial data points in the source databases; (b) molecule PubChem CID and SID; (c) molecule SMILES and InChI; (d) target UniProt ID;^69^ (e) bioactivity type (e.g., *K*_i_, *K*_d_, EC_50_, IC_50_, others); (f) bioactivity relation (e.g., “=”, “<”, “≤”); and (g) bioactivity value, including the mean, median, standard deviation, and count. These comprehensive data sets facilitate a robust platform for the evaluation and analysis of CPI and AC performances.

#### Multistep Data Cleaning, Processing, and Integrating Workflow

The workflow for cleaning and integrating bioactivity data from EquiVS and Papyrus sources aimed to ensure high data quality and is summarized in Figure S9. This multistep procedure involves specific filtering steps applied differently to each data source to refine the bioactivity data available for analysis. Regarding EquiVS data processing, the workflow contains the following: (a) unit filtering: bioactivity data points measured in units that can be converted to the negative log scale (e.g., *K*_i_, EC_50_) were selected to maintain consistency in quantification; (b) activity-type filtering: only data points corresponding to specific activity types (*K*_i_, *K*_d_, EC_50_, IC_50_, potency) were retained; (c) conflict value filtering: data involving the same targets and molecules but displaying divergent bioactivity values were scrutinized for discrepancies. Data points showing variations greater than 1 negative log­(more than a 10-fold difference) were excluded. Remaining conflicts were resolved by averaging the values to provide corrected bioactivities; (d) protein ID mapping: conversion of protein HGNC IDs to available UniProt IDs was required to integrate external structural information. Data points lacking a corresponding UniProt ID were discarded. Regarding Papyrus data processing, the workflow contains the following: (a) relation-type filtering: only data points with relation types “=” or “≤” were retained to ensure the reliability of bioassay measurements. Notably, a significant portion of Papyrus data (∼55 M entries) was excluded due to missing relation annotations. (b) Activity-type filtering: data points classified under activity types *K*_i_, *K*_d_, EC_50_, and IC_50_ were kept. (c) Activity standard deviation filtering: a criterion of standard deviations less than 0.1 was applied to select bioactivity data that demonstrated a high measurement consistency. Upon screening the preliminary bioactivity data, a molecule structure checking process was implemented using MolVS^70^ and RDKit.^71^ This step ensured the normalization of SMILES representations and the elimination of invalid and duplicate data through several key procedures: (a) functional group normalization; (b) charge reintegration; (c) metal bond breaking; (d) competitive reionization; (e) tautomer enumeration and canonicalization; (f) neutralization of charges; (g) stereochemistry standardization; (h) salt and solvent fragment filtering; (i) fragment, isotope, charge, tautomer, or stereochemistry-insensitive parent structure creation; and (j) unusual characteristics validation. Following molecule normalization, the protein sequence and structural data were validated and collected. Wildtype protein sequences were sourced from the UniProt database, facilitating the generation of protein structures and the construction of sequence-based CPI methods. Given the limitations of experimentally determined structures in the PDB database, which may not crystallize all residue conformations and could have missing pockets, we utilized AlphaFold 2 ^72^ to generate comprehensive protein structures from amino acid sequences. This approach ensured that full-length crystallized protein structures were available for CPI model training.

After molecule and protein structure representations were standardized, duplicate and conflicting CPI data entries were removed using the conflict value filtering strategy initially applied to EquiVS data. These filtering strategies were conducted for reducing measurement noise and enhancing the reliability of the data set used in our model training and the subsequent analysis.

#### Bioassay Selection

Based on the definitions of activity cliff from previous studies,^27,28^ specific bioactivity data types including *K*_i_, *K*_d_, EC_50_, and IC_50_ were selected for comprehensive AC effect estimation. Other assay data types were excluded due to the unreliability of their experimental noise levels in supporting a consistent AC analysis. The constructed *K*_i_, *K*_d_, EC_50_, and IC_50_ benchmark data sets were used for subsequent model training and comparison.

#### Activity Cliff Identification and Labeling

Activity cliffs were identified using the criteria established in MoleculeACE.^31^ Initially, comprehensive structural similarities between molecule pairs targeting the same protein were calculated. This included combining the Tanimoto coefficient for all-atom molecule fingerprints and scaffold molecule fingerprints along with the Levenshtein distance of SMILES sequences. Similar molecule pairs were identified if at least one of the three similarity methods produced a score greater than 0.9. Subsequent bioactivity value checks filtered out those structurally similar pairs that exhibited a negative log bioactivity value difference of >2 (equivalent to a 100-fold difference in nM units). Pairs meeting these criteria were labeled as AC data, while others were classified as non-AC data.

#### Data Splitting and Benchmark Data Set Construction

For the construction of benchmark data sets, an AC-specific data splitting approach was employed as described in MoleculeACE.^31^

This method was designed by MoleculeACE to ensure (a) a proportional representation of the number of activity cliff compounds in the training and test sets (to avoid an over/underestimation of their effect on the performance) and (b) preserving structural similarity between training and test molecules, as previously suggested.^73^ To this end, for each data set, molecules were clustered based on substructure similarity using spectral clustering (50) on extended connectivity fingerprints (ECFPs). For each cluster, molecules were split into a training (80%) and test sets (20%) by stratified random sampling using their AC label. This strategy ensures that even if all partners in an AC pair end up in the test set, structurally similar molecules are likely still present in the training set, maintaining the representation of local chemical space. For each target in the CPI2M-main data set, we applied AC-specific splitting to its available data. We then combined the resulting per-target training and testing subsets across all targets to assemble the final CPI2M-main training and CPI2M-main validation sets, thereby preserving the AC-specific split as we move from target-specific to general CPI settings.

### GGAP-CPI Architecture

Figure 1 depicts the model architecture of GGAP-CPI, which consists of a ligand encoder, a protein encoder, a multihead cross-attention pooling module, and a feed-forward neural network decoder. We introduce each module in the GGAP-CPI in detail.

#### Ligand Encoder

Pretrained models were proven to show highly competitive performances in molecular property prediction tasks. In our study, a pretrained molecule encoder (knowledge graph-enhanced molecular contrastive learning with functional prompt, KANO^40^) was introduced as the ligand encoder to learn advanced atom-level and molecule-level hidden embeddings. Concretely, KANO is a *L*-layered C-MPNN-based graph neural network^74^ which is pretrained based on a chemical element knowledge graph and contrastive learning. Regarding the L-layered C-MPNN process, given an atom node *v* and its node set $N_{v}$ for a molecular graph G , an intermediate message vector *M* ^(*l*)^(*v*) in *l*th layer is obtained by

1 $$M^{\left(l\right)}\left(v\right)=\sum_{u\in N_{v}}H^{\left(l-1\right)}\left(e_{u,v}\right)⊙\max\left(\sum_{u\in N_{v}}H^{\left(l-1\right)}\left(e_{u,v}\right)\right)$$

where *e*_*u*,*v*_ is the edge vector between *v* and its neighbor node *u* and ⊙ is an element-wise multiplication operator. The current nodes’ representation *H* ^(*l*)^(*v*) is obtained by concatenating the message vector and previous representation:

2 $$H^{\left(l\right)}\left(v\right)=ReLU\left(W^{\left(l\right)}Concat\left(H^{\left(l-1\right)}\left(v\right),M^{\left(l\right)}\left(v\right)\right)\right)$$

where *W* ^(*l*)^ is the trainable parameter matrix. Meanwhile, the edge representations are updated simultaneously to enhance the interactions between the atoms and chemical bonds. The message of the edge *e*_*u*,*v*_ is extracted by subtracting its inverse edge information from *H* ^(*l*)^(*v*):

3 $$M^{\left(l\right)}\left(e_{u,v}\right)=H^{\left(l\right)}\left(v\right)-H^{\left(l-1\right)}\left(e_{v,u}\right)$$

Given the initial edge feature *H* ^(0)^(*e*_*v*,*u*_), the updating of edge representation *H* ^(*l*)^(*e*_*v*,*u*_) is formulated as

4 $$H^{\left(l\right)}\left(e_{v,u}\right)=ReLU\left(H^{\left(0\right)}\left(e_{v,u}\right)+WM^{\left(l\right)}\left(e_{v,u}\right)\right)$$

After *L* iterations, an extra message passing and aggregating process is used for final message *M*(*v*) and node *H*_atom_(*v*) representation learning:

5 $$M\left(v\right)=\sum_{u\in N_{v}}H^{\left(L\right)}\left(e_{u,v}\right)⊙\max\left(\sum_{u\in N_{v}}H^{\left(L\right)}\left(e_{u,v}\right)\right)$$

6 $$H_{atom}\left(v\right)=ReLU\left(WConcat\left(M\left(v\right),H^{\left(L\right)}\left(v\right),H^{\left(0\right)}\left(e_{v}\right)\right)\right)$$

Finally, a readout GRU (gated recurrent unit) with a sum operator is applied to get ligand graph-level embedding *H*_lig_:

7 $$H_{lig}=\sum_{v\in N_{G}}GRU\left(H_{atom}\left(v\right)\right)$$

#### Protein Encoder

Protein language models (e.g., ESM-2,^41^ AlphaFold 2 ^72^) have promoted the monomer protein structure prediction to experiment-level accuracy. Many recent studies have proven the notable contribution of protein embeddings from protein language models to the protein–ligand binding affinity prediction task. Therefore, we adopted ESM-2 (ESM-2_t33_650M_UR50D) to generate residue embeddings. Then, inspired by other protein function predictions,^75^ we considered introducing protein graphs with multiple residue contact types to acquire higher-order protein representations by a graph convolution process. Specifically, 7 residue contact types (peptide bond, hydrogen bond interaction, disulfide interaction, ionic interaction, aromatic interaction, aromatic sulfur interaction, and cation–pi interaction) were considered for adjacency matrix generation for protein graphs using Graphein.^76^ Then, given the initial protein residue embedding *H*_res_^(0)^ and protein graph adjacency matrix *A*_prot_, a L-layered graph convolutional network (GCN)^77^ was constructed for residue-level and protein-level embedding generation. For the *l*th layer, the GCN process can be formulated as

8 $$H_{res}^{\left(l\right)}=ReLU\left({\mathrm{D̂}}_{prot}^{-1/2}{Â}_{prot}{\mathrm{D̂}}_{prot}^{-1/2}H_{res}^{\left(l-1\right)}W^{\left(l\right)}\right)$$

After the residue embeddings were obtained, an average pooling was used to aggregate the node-level embeddings to graph-level embeddings:

9 $$H_{prot}=\frac{1}{N_{res}}\sum_{i=1}^{N_{res}}H_{res\left(i\right)}^{\left(L\right)}$$

#### Multihead Cross-Attention Pooling

After the input ligand and protein structures were encoded as multiscale embeddings, a multihead cross-attention pooling method was designed to aggregate the current four embeddings (*H*_atom_, *H*_lig_, *H*_res_, and *H*_prot_) to protein–ligand complex embedding with hierarchical structure representation. A cross-attention strategy has been applied to various biomedical tasks for cross-modality representation aggregation.^78–80^ Compared to self-attention, a more well-recognized attention-based mechanism, cross-attention sets the embedding from one modality/source as the query vector and the embedding from the other modality/source as the key and value vectors execute the attention matrix calculation and weighted aggregation. The matrix computing and aggregating flow regarding the multihead cross-attention pooling is shown in Figure S10.

Specifically, given the number of attention heads as *N*_attn_ for the *n*th head and the feature dimension as *d*_h_, the ligand atomic embedding was defined as the query vector $\left(Q_{atom}^{\left(n\right)}\in R^{N_{atom}\times d_{h}/N_{attn}}\right)$ that underwent a linear transformation for dimension alignment, while the protein residual embedding was defined as the key $\left(K_{res}^{\left(n\right)}\in R^{N_{res}\times d_{h}/N_{attn}}\right)$ and value vectors $\left(V_{res}^{\left(n\right)}\in R^{N_{res}\times d_{h}/N_{attn}}\right)$ with a linear transformation process. The previous definition can be formulated as

10 $$Q_{atom}^{\left(n\right)}=H_{atom}W_{Q}^{\left(n\right)}+b_{Q}^{\left(n\right)},K_{res}^{\left(n\right)}=H_{res}W_{K}^{\left(n\right)}+b_{K}^{\left(n\right)},V_{res}^{\left(n\right)}=H_{res}W_{V}^{\left(n\right)}+b_{V}^{\left(n\right)}$$

where *W*_*Q*_^(*n*)^, *W*_*K*_^(*n*)^, *W*_*V*_^(*n*)^, *b*_*Q*_^(*n*)^, *b*_*K*_^(*n*)^, and *b*_*V*_^(*n*)^ are trainable parameter matrices. Then, a normalized attention matrix $A^{\left(n\right)}\in R^{N_{atom}\times N_{res}}$ in the *n*th head was calculated using an inner product of *Q*_atom_^(*n*)^ and *K*_res_^(*n*)^ divided by a dimensional scaling factor $\sqrt{\frac{d_{h}}{N_{attn}}}$ , which is formulated as

11 $$A^{\left(n\right)}=Softmax\left(\frac{Q_{atom}^{\left(n\right)}{K_{res}^{\left(n\right)}}^{T}}{\sqrt{\frac{d_{h}}{N_{attn}}}}\right)$$

The calculated attention matrix reflects the dynamic pairwise attention and importance weights for ligand atoms and protein residues. Therefore, we further applied it for weighted aggregation to obtain complex embedding. The aggregation process to acquire the output complex embedding $H_{comp}\in R^{1\times d_{h}}$ can be formulated as

12 $$H_{comp}={⊕}_{n=1}^{N_{attn}}average\left(A^{\left(n\right)}V_{res}^{\left(n\right)}\right)$$

where the operator “⊕” represents the concatenation process. The final CPI complex embedding is formed by concatenating the molecular graph embedding, protein graph embedding, and complex embeddings as a comprehensive aggregation of multiple pooling outputs:

13 $$H_{CPI}=H_{lig}⊕H_{comp}⊕H_{prot}$$

#### Feed-Forward Decoder

The CPI complex embedding generated from the multihead cross-attention pooling block is fed into a two-layered feed-forward decoder for final binding affinity/bioactivity prediction, which is formulated as

14 $$y=\left(ReLU\left(H_{CPI}W^{\left(0\right)}+b^{\left(0\right)}\right)\right)W^{\left(1\right)}+b^{\left(0\right)}$$

where *y* is the output prediction, while *W* and *b* are trainable parameter matrixes.

### Integrated Bioactivity Learning

To fully leverage our CPI2M data and train competitive bioactivity prediction models, we integrated bioactivity data with different activity types into a single data set and applied an ensemble learning approach for training the GGAP-CPI model. Specifically, we compiled all available bioactivity data in the training set (*N* = 1,185,824) into one data set. We then performed a standard 5-fold cross-validation to train 5 GGAP-CPI models.

To further optimize the ensemble learning process, particularly in terms of bioactivity data quality, we conducted an equal sampling of K_i_ data from the validation set based on its bioactivity distribution. This sampled data was used as the validation set for model selection, with the aim of retaining the most robust GGAP-CPI model, capable of accurate prediction on high-quality and long-tail distributed bioactivity data. Then, another 5-fold cross-validation was conducted to train 5 GGAP-CPI models.

Once the model training was completed, all 10 trained models, collectively referred to as GGAP-CPI, were used for subsequent predictions and evaluations by averaging the predictions. The process workflow for integrated bioactivity learning is shown in Figure S11.

### Experimental Setup

#### Baseline Setup

Building on previous studies that quantified the activity-cliff effect using methods that exclusively consider ligand structures, we developed comprehensive model comparisons, incorporating both target-specific molecular property prediction models and general CPI models as baseline methods.

For target-specific approaches, we introduced a diverse set of models: (a) machine learning methods with ligand ECFP4-2048 bit encoding: Support Vector Machine (SVM), Random Forest (RF), Gradient Boosting Machine (GBM), K-Nearest Network (KNN), and Multilayer Perceptron (MLP); (b) graph-based deep learning methods with ligand graphs: Graph Convolutional Network (GCN^77^), Graph Attention Network (GAT^81^), Message Passing Neural Network (MPNN^82^), Attentive Fingerprint (AFP^83^), and KANO;^40^ (c) sequence-based methods with augmented ligand SMILES: Convolutional Neural Network (CNN) and Transformer.^84^ The CPI2M data set was partitioned by protein into target-specific subsets to facilitate model training tailored to specific targets. Predicted bioactivities from the testing sets of each subset were aggregated and analyzed to ensure consistency with the CPI model predictions.

For CPI approaches integrating both ligand and protein inputs, we employed (a) machine learning methods: ECFP-ESM-RF and ECFP-ESM-GBM; and (b) deep learning methods: DeepDTA,^85^ GraphDTA,^86^ HyperAttentionDTI,^87^ PerceiverCPI,^78^ and KANO-ESM. The machine learning methods utilized featurization based on ECFP embeddings for ligands coupled with ESM-2 embeddings for proteins. KANO-ESM, specifically, was constructed with a trainable KANO encoder for ligand encoding, averaged ESM-2 residue embeddings for protein encoding, and a feed-forward decoder for CPI bioactivity prediction. Other deep learning methods were applied as per protocols established in their respective original studies.

#### Hyperparameter Setting

For the GGAP-CPI model, hyperparameters were determined based on guidelines from the original KANO study^40^ and further refined through empirical testing. The configuration included the following: the hidden dimension size for the ligand and protein encoders is 300; the number of C-MPNN layers in the ligand encoder and GCN layers in the protein encoder is 1; the number of attention heads in multihead cross-attention pooling is 5; and the dropout rate is 0.1. GGAP-CPI was optimized with the optimization function of mean square error (MSE) loss, a batch size of 256, a training epoch of 100, and a learning rate of 1 × 10^–4^. The overall number of trainable parameters in GGAP-CPI is 3.2 M. For fine-tuning GGAP-CPI on downstream data sets, we perform a full training cycle in which all model parameters are updated.

For other baseline methods, hyperparameters were adopted as per the specifications detailed in their respective original studies, ensuring consistency with published results. Target-specific methods and machine learning-based CPI models replicated the same settings as reported in the MoleculeACE study.^31^ In all, the hyperparameter setting facilitated fair and consistent comparisons across models by maintaining general parameter settings without data set-specific adjustments.

### Evaluation Metrics

To assess the performance of GGAP-CPI and other baselines, we employed several metrics, addressing both the general efficacy and the specific performance concerning AC data. For overall performance, the root-mean-square error (RMSE), Pearson’s correlation coefficient (PCC), and Spearman’s correlation coefficient (SRCC) were computed on the bioactivity values (i.e., *K*_i_ or EC_50_) as follows:

15 $$RMSE=\sqrt{\frac{\sum_{i=1}^{n}{\left({ŷ}_{i}-y_{i}\right)}^{2}}{n}}$$

16 $$PCC=\frac{\sum_{i=1}^{n}\left(y_{i}-\mathrm{y̅}\right)\left(\hat{y_{i}\;}-\overset{®}{\hat{y}}\right)}{\sqrt{\sum_{i=1}^{n}{\left(y_{i}-\mathrm{y̅}\right)}^{2}\sum_{i=1}^{n}{\left(\hat{y_{i}\;}-\overset{®}{\hat{y}}\right)}^{2}}}$$

17 $$SRCC=\frac{\sum_{j=1}^{n}\left(R\left(y_{j}\right)-\overset{®}{R\left(y\right)}\right)\left(R\left({ŷ}_{j}\right)-\overset{®}{R\left(ŷ\right)}\right)}{\sqrt{\sum_{j=1}^{n}{\left(R\left(y_{j}\right)-\overset{®}{R\left(y\right)}\right)}^{2}\sum_{j=1}^{n}{\left(R\left({ŷ}_{j}\right)-\overset{®}{R\left(ŷ\right)}\right)}^{2}}}$$

where $\hat{y_{i}\;}$ is the predicted bioactivity of the *i*th end point, while *y*_*i*_ is the true bioactivity value. *R*(*y*_*j*_) denotes the rank of the true bioactivity value *y*_*j*_ for the *j*th end point, and $R\left({ŷ}_{j}\right)$ denotes the rank of the predicted bioactivity value ${ŷ}_{j}$ for the *j*th end point. $\overset{®}{R\left(y\right)}$ and $\overset{®}{R\left(ŷ\right)}$ represent the mean ranks of the true and predicted bioactivity values, respectively. *n* represents the number of data being considered.

For AC-specific model performance comparison, followed by the metric RMSE_cliff_ proposed by MoleculeACE,^31^ which incorporated the RMSE value for AC molecules as the standard criterion, we adopted and expanded it to PCC_cliff_ and SRCC_cliff_ as two newly enrolled metrics. The overall calculations of these AC-specific metrics are totally the same as those in eqs 15–17. The only difference is they are calculated only on AC molecules rather than all molecules.

For the virtual screening assessment, we employed metrics to calculate the scoring, ranking, and screening power. For scoring power, PCC is used as the metric, which is equivalent to eq 16. For ranking power, the Spearman’s rank correlation coefficient (SRCC) is adopted as the same as eq 17. For screening power, the enrichment factor (EF_1%_) among the 1% of the top-ranked ligands is adopted. The EF_1%_ is calculated as follows

18 $$EF_{1\%}=\frac{NTB_{1\%}}{NTB_{total}\times 1\%}$$

*n* indicates the number of data. NTB_1%_ is the number of true binders observed among the 1% of top-ranked candidates selected by a given scoring function. NTB_total_ is the total number of true binders for the given target protein

## Supplementary Material



## Data Availability

The MoleculeACE, CASF-2016, MerckFEP, DUD-E, DEKOIS-v2, and LIT-PCBA benchmark data were downloaded from the following sources: https://github.com/molML/MoleculeACE, http://www.pdbbind.org.cn/casf.php, https://github.com/MCompChem/fep-benchmark, https://dude.docking.org/, http://www.dekois.com, and https://drugdesign.unistra.fr/LIT-PCBA/, respectively. The processed CPI2M benchmark data are publicly available at https://zenodo.org/records/15789422. The source code of the GGAP-CPI and CPI2M data processing procedure is publicly available at https://github.com/gu-yaowen/GGAP-CPI.

## Notes

Overlaps in CPI2M; PCA feature deduction visualization for CPI2M; comparison of overall general Pearson’s correlation coefficient (PCC) and AC-specific PCC (PCC_cliff_) for GGAP-CPI, target-specific baselines, and CPI baselines on the internal CPI2M-main validation sets; comparison of overall general Spearman’s correlation coefficient (SRCC) and AC-specific SRCC (SRCC _cliff_) for GGAP-CPI, target-specific baselines, and CPI baselines on the internal CPI2M-main validation sets; comparison of overall general AC-specific performances for GGAP-CPI (rose red) and CPI baselines (steel blue) on the internal CPI2M-few test sets (*K*_i_, *K*_d_, EC_50_, IC_50_), with diagonal hatching denoting the best-performing model and vertical-line hatching denoting the second-best: overall RMSE_cliff_, overall PCC_cliff_, and overall SRCC_cliff_; comparison of overall general performance metrics—root-mean-square error (RMSE), Pearson’s correlation coefficient (PCC), and Spearman’s rank correlation coefficient (SRCC)—for GGAP-CPI and CPI baselines on the external CPI2M-few-cold-drug test sets; subset MAE vs uncertainty threshold for GGAP-CPI on the CPI2M-few external test sets; overall RMSE of GGAP-CPI on the CPI2M-main internal validation sets as a function of training data fraction (5%, 10%, 20%, and 100%); data collection, processing, and integrating workflow for the CPI2M benchmark, with EquiVS and Papyrus data collected with about 70 M measurements, a multistep filtering and processing strategy used to screen high-quality CPI data from two sources, and then the integrated CPI data after duplicate data removal and protein structure availability check categorized based on the activity types to generate six subsets (*K*_i_, *K*_d_, EC_50_, IC_50_, potency, and percentage of activity) as the final candidates for CPI2M; computing flow in the multihead cross-attention pooling module to generate CPI complex embedding from ligand and protein embeddings, with the operator “⊕” representing concatenation and “⊗” representing inner product with linear transformation and nonlinear activation; integrated bioactivity learning process for GGAP-CPI; target-specific performance results of general and AC-specific metrics (RMSE, RMSE_cliff_, PCC, PCC_cliff_, SRCC, SRCC_cliff_) for GGAP-CPI and baselines on the internal CPI2M-main-*K*_i_ validation sets; target-specific performance results of general and AC-specific metrics for GGAP-CPI and baselines on the internal CPI2M-main-*K*_d_ validation sets; target-specific performance results of general and AC-specific metrics for GGAP-CPI and baselines on the internal CPI2M-main-EC_50_ validation sets; target-specific performance results of general and AC-specific metrics for GGAP-CPI and baselines on the internal CPI2M-main-IC_50_ validation sets; target-specific performance results of general and AC-specific metrics (RMSE, RMSE_cliff_, PCC, PCC_cliff_, SRCC, SRCC_cliff_) for GGAP-CPI and baselines on the internal CPI2M-few-*K*_i_ test sets; target-specific performance results of general and AC-specific metrics for GGAP-CPI and baselines on the internal CPI2M-few-*K*_d_ test sets; target-specific performance results of general and AC-specific metrics for GGAP-CPI and baselines on the internal CPI2M-few-EC_50_ test sets; target-specific performance results of general and AC-specific metrics for GGAP-CPI and baselines on the internal CPI2M-few-IC_50_ test sets; target-specific performance results of general and AC-specific metrics for GGAP-CPI and baselines on the internal MoleculeACE-*K*_i_ validation sets; target-specific performance results of general and AC-specific metrics for GGAP-CPI and baselines on the internal MoleculeACE-EC_50_ validation sets; overall performance results of general and AC-specific metrics for GGAP-CPI on data from the top 5 protein families in the CPI2M-main internal validation sets; overall performance results of general and AC-specific metrics for GGAP-CPI on data from different molecular weight intervals in the CPI2M-main interval validation sets; overall performance on the CASF-2016 data set; overall ranking power on the Merck FEP data set with AlphaFold2-predicted structures used for inference; overall ranking power on the Merck FEP data set with crystal structures used for inference; overall EF1% on the LIT-PCBA data set with AlphaFold2-predicted structures used for inference; overall EF1% on the LIT-PCBA data set with crystal structures used for inference; and inference times for GGAP-CPI, its five variants, and PerceiverCPI on one target (P42336) with 1000 ligands (PDF)

## References

[1] Yang C., Chen E. A., Zhang Y. (2022). Protein–ligand docking in
the machine-learning era. Molecules.

[2] Yang C., Zhang Y. (2022). Delta machine learning to improve
scoring-ranking-screening performances
of protein–ligand scoring functions. J. Chem. Inf. Model..

[3] Yang C., Zhang Y. (2021). Lin_F9: a
linear empirical scoring function for protein–ligand
docking. J. Chem. Inf. Model..

[4] Gu Y., Li J., Kang H., Zhang B., Zheng S. (2023). Employing
Molecular
Conformations for Ligand-Based Virtual Screening with Equivariant
Graph Neural Network and Deep Multiple Instance Learning. Molecules.

[5] Pan X., Wang H., Zhang Y., Wang X., Li C., Ji C., Zhang J. Z. (2022). AA-score:
a new scoring function based on amino acid-specific
interaction for molecular docking. J. Chem.
Inf. Model..

[6] You Y., Shen Y. (2022). Cross-modality and
self-supervised protein embedding for compound–protein
affinity and contact prediction. Bioinformatics.

[7] Xia S., Gu Y., Zhang Y. (2025). Normalized
Protein–Ligand Distance Likelihood
Score for End-to-End Blind Docking and Virtual Screening. J. Chem. Inf. Model..

[8] Wang R., Fang X., Lu Y., Yang C.-Y., Wang S. (2005). The PDBbind
database: methodologies and updates. J. Med.
Chem..

[9] Gaulton A., Bellis L. J., Bento A. P., Chambers J., Davies M., Hersey A., Light Y., McGlinchey S., Michalovich D., Al-Lazikani B. (2012). ChEMBL: a large-scale
bioactivity database for drug discovery. Nucleic
Acids Res..

[10] Liu T., Lin Y., Wen X., Jorissen R. N., Gilson M. K. (2007). BindingDB: a web-accessible
database of experimentally determined protein–ligand binding
affinities. Nucleic Acids Res..

[11] Volkov M., Turk J.-A., Drizard N., Martin N., Hoffmann B., Gaston-Mathé Y., Rognan D. (2022). On the frustration to predict binding
affinities from protein–ligand structures with deep neural
networks. J. Med. Chem..

[12] Trott O., Olson A. J. (2010). AutoDock Vina: improving the speed
and accuracy of
docking with a new scoring function, efficient optimization, and multithreading. J. Comput. Chem..

[13] McNutt A. T., Francoeur P., Aggarwal R., Masuda T., Meli R., Ragoza M., Sunseri J., Koes D. R. (2021). GNINA 1.0: molecular
docking with deep learning. J. Cheminf..

[14] Ballester P. J., Mitchell J. B. (2010). A machine learning approach to predicting
protein–ligand
binding affinity with applications to molecular docking. Bioinformatics.

[15] Wang C., Zhang Y. (2017). Improving scoring-docking-screening powers of protein–ligand
scoring functions using random forest. J. Comput.
Chem..

[16] Lam H. Y. I., Guan J. S., Ong X. E., Pincket R., Mu Y. (2024). Protein language
models are performant in structure-free virtual screening. bioRxiv.

[17] Wang S., Jiang M., Zhang S., Wang X., Yuan Q., Wei Z., Li Z. (2021). MCN-CPI: multiscale
convolutional network for compound–protein
interaction prediction. Biomolecules.

[18] Feng B., Liu Z., Huang N., Xiao Z., Zhang H., Mirzoyan S., Xu H., Hao J., Xu Y., Zhang M. (2024). A bioactivity
foundation model using pairwise meta-learning. Nat. Mach. Intell..

[19] Yan J., Ye Z., Yang Z., Lu C., Zhang S., Liu Q., Qiu J. (2024). Multi-task bioassay
pre-training for protein-ligand binding affinity
prediction. Briefings Bioinf..

[20] Yin Y., Lam H. Y. I., Mu Y., Li H. Y., Kong A. W.-K. (2024). Advancing
Bioactivity Prediction through Molecular Docking and Self-Attention. IEEE J. Biomed. Health Inform..

[21] Gorantla R., Gema A. P., Yang I. X., Serrano-Morrás A. ´., Suutari B., Jiménez J. J., Mey A. S. (2024). Learning Binding
Affinities via Fine-tuning of Protein and Ligand Language Models. bioRxiv.

[22] Cer R. Z., Mudunuri U., Stephens R., Lebeda F. J. (2009). IC 50-to-K
i: a
web-based tool for converting IC 50 to K i values for inhibitors of
enzyme activity and ligand binding. Nucleic
Acids Res..

[23] Kalliokoski T., Kramer C., Vulpetti A., Gedeck P. (2013). Comparability of mixed
IC_50_ data–a statistical analysis. PLoS One.

[24] Hernández-Garrido C. A., Sánchez-Cruz N. (2023). Experimental
Uncertainty in Training Data for Protein-Ligand
Binding Affinity Prediction Models. Artif. Intell.
Life Sci..

[25] Landrum G. A., Riniker S. (2024). Combining IC_50_ or K i Values from Different
Sources Is a Source of Significant Noise. J.
Chem. Inf. Model..

[26] Theisen R., Wang T., Ravikumar B., Rahman R., Cichońska A. (2024). Leveraging
multiple data types for improved compound-kinase bioactivity prediction. Nat. Commun..

[27] Stumpfe D., Hu Y., Dimova D., Bajorath J. (2014). Recent Progress in Understanding
Activity Cliffs and Their Utility in Medicinal Chemistry. J. Med. Chem..

[28] Stumpfe D., Bajorath J. (2012). Exploring Activity
Cliffs in Medicinal Chemistry. J. Med. Chem..

[29] Park J., Sung G., Lee S., Kang S., Park C. (2022). ACGCN: Graph
Convolutional Networks for Activity Cliff Prediction between Matched
Molecular Pairs. J. Chem. Inf. Model..

[30] Yang, H. ; Yao, Q. ; Kwok, J. Curriculum-aware Training for Discriminating Molecular Property Prediction Models. In Thirteenth International Conference on Learning Representations.

[31] van
Tilborg D., Alenicheva A., Grisoni F. (2022). Exposing the Limitations
of Molecular Machine Learning with Activity Cliffs. J. Chem. Inf. Model..

[32] Zhang Z., Zhao B., Xie A., Bian Y., Zhou S. (2023). Activity cliff
prediction: Dataset and benchmark. arXiv.

[33] Chen H., Vogt M., Bajorath J. (2022). DeepAC–conditional
transformer-based
chemical language model for the prediction of activity cliffs formed
by bioactive compounds. Digital Discovery.

[34] Chen X., Yu D., Zhao L., Liu F. (2025). ACES-GNN: Can Graph Neural Network
Learn to Explain Activity Cliffs?. Digital Discovery.

[35] Dimova D., Heikamp K., Stumpfe D., Bajorath J. (2013). Do medicinal chemists
learn from activity cliffs? A systematic evaluation of cliff progression
in evolving compound data sets. J. Med. Chem..

[36] Yin Y., Hu H., Yang J., Ye C., Goh W. W. B., Kong A. W.-K., Wu J. (2024). OLB-AC: toward optimizing ligand bioactivities through
deep graph learning and activity cliffs. Bioinformatics.

[37] Wedlake A. J., Folia M., Piechota S., Allen T. E., Goodman J. M., Gutsell S., Russell P. J. (2020). Structural
alerts and random forest
models in a consensus approach for receptor binding molecular initiating
events. Chem. Res. Toxicol..

[38] Stumpfe D., Bajorath J. (2012). Exploring activity
cliffs in medicinal chemistry: miniperspective. J. Med. Chem..

[39] Stumpfe D., Hu H., Bajorath J. (2019). Evolving Concept
of Activity Cliffs. ACS Omega.

[40] Fang Y., Zhang Q., Zhang N., Chen Z., Zhuang X., Shao X., Fan X., Chen H. (2023). Knowledge
graph-enhanced
molecular contrastive learning with functional prompt. Nat. Mach. Intell..

[41] Lin Z., Akin H., Rao R., Hie B., Zhu Z., Lu W., Smetanin N., Verkuil R., Kabeli O., Shmueli Y. (2023). Evolutionary-scale prediction
of atomic-level protein structure with
a language model. Science.

[42] Su M., Yang Q., Du Y., Feng G., Liu Z., Li Y., Wang R. (2019). Comparative Assessment of Scoring Functions: The CASF-2016
Update. J. Chem. Inf. Model..

[43] Schindler C. E., Baumann H., Blum A., Böse D., Buchstaller H.-P., Burgdorf L., Cappel D., Chekler E., Czodrowski P., Dorsch D. (2020). Large-scale assessment
of binding free energy calculations in active drug discovery projects. J. Chem. Inf. Model..

[44] Mysinger M. M., Carchia M., Irwin J. J., Shoichet B. K. (2012). Directory of useful
decoys, enhanced (DUD-E): better ligands and decoys for better benchmarking. J. Med. Chem..

[45] Bauer M. R., Ibrahim T. M., Vogel S. M., Boeckler F. M. (2013). Evaluation and optimization
of virtual screening workflows with DEKOIS 2.0–a public library
of challenging docking benchmark sets. J. Chem.
Inf. Model..

[46] Tran-Nguyen V.-K., Jacquemard C., Rognan D. (2020). LIT-PCBA: an unbiased data set for
machine learning and virtual screening. J. Chem.
Inf. Model..

[47] Hunter S., Apweiler R., Attwood T. K., Bairoch A., Bateman A., Binns D., Bork P., Das U., Daugherty L., Duquenne L. (2009). InterPro: the integrative
protein signature
database. Nucleic Acids Res..

[48] Jiang D., Wu Z., Hsieh C. Y., Chen G., Liao B., Wang Z., Shen C., Cao D., Wu J., Hou T. (2021). Could graph
neural networks learn better molecular representation for drug discovery?
A comparison study of descriptor-based and graph-based models. J. Cheminf..

[49] Moon S., Zhung W., Yang S., Lim J., Kim W. Y. (2022). PIGNet:
a physics-informed deep learning model toward generalized drug-target
interaction predictions. Chem. Sci..

[50] Yang C., Zhang Y. (2022). Delta Machine Learning to Improve Scoring-Ranking-Screening Performances
of Protein-Ligand Scoring Functions. J. Chem.
Inf. Model..

[51] Nguyen T., Le H., Quinn T. P., Nguyen T., Le T. D., Venkatesh S. (2021). GraphDTA:
predicting drug–target binding affinity with graph neural networks. Bioinformatics.

[52] Jiménez J., Skalic M., Martinez-Rosell G., De Fabritiis G. K. (2018). deep: protein–ligand
absolute binding affinity prediction via 3d-convolutional neural networks. J. Chem. Inf. Model..

[53] Shen C., Zhang X., Deng Y., Gao J., Wang D., Xu L., Pan P., Hou T., Kang Y. (2022). Boosting Protein-Ligand
Binding Pose Prediction and Virtual Screening Based on Residue-Atom
Distance Likelihood Potential and Graph Transformer. J. Med. Chem..

[54] Guichaoua G., Pinel P., Hoffmann B., Azencott C.-A., Stoven V. (2024). Drug–target
interactions prediction at scale: The komet algorithm with the lcidb
dataset. J. Chem. Inf. Model..

[55] Singh R., Sledzieski S., Bryson B., Cowen L., Berger B. (2023). Contrastive
learning in protein language space predicts interactions between drugs
and protein targets. Proc. Natl. Acad. Sci.
U.S.A..

[56] Liao, Z. ; You, R. ; Huang, X. ; Yao, X. ; Huang, T. ; Zhu, S. DeepDock: enhancing ligand-protein interaction prediction by a combination of ligand and structure information. IEEE International Conference on Bioinformatics and Biomedicine (BIBM), 2019, pp 311–317.

[57] Moon S., Hwang S.-Y., Lim J., Kim W. Y. (2024). PIGNet2: a versatile
deep learning-based protein–ligand interaction prediction model
for binding affinity scoring and virtual screening. Digital Discovery.

[58] Li H., Lu G., Sze K.-H., Su X., Chan W.-Y., Leung K.-S. (2021). Machine-learning
scoring functions trained on complexes dissimilar to the test set
already outperform classical counterparts on a blind benchmark. Briefings Bioinf..

[59] Meli R., Anighoro A., Bodkin M. J., Morris G. M., Biggin P. C. (2021). Learning
protein-ligand binding affinity with atomic environment vectors. J. Cheminf..

[60] Lakshminarayanan, B. ; Pritzel, A. ; Blundell, C. Simple and scalable predictive uncertainty estimation using deep ensembles. In Advances in neural information processing systems, 2017; 30

[61] Xia S., Zhang D., Zhang Y. (2023). Multitask Deep Ensemble Prediction
of Molecular Energetics in Solution: From Quantum Mechanics to Experimental
Properties. J. Chem. Theory Comput..

[62] Pan X., Gu Y., Zhou W., Zhang Y. (2025). Enhancing Transthyretin
Binding Affinity
Prediction with a Consensus Model: Insights from the Tox24 Challenge. Chem. Res. Toxicol..

[63] Béquignon O. J.
M., Bongers B. J., Jespers W., Ijzerman A. P., van der
Water B., van Westen G. J. P. (2023). Papyrus: a large-scale curated dataset
aimed at bioactivity predictions. J. Cheminf..

[64] Kim S., Chen J., Cheng T., Gindulyte A., He J., He S., Li Q., Shoemaker B. A., Thiessen P. A., Yu B. (2023). PubChem 2023 update. Nucleic Acids Res..

[65] Škuta C., Southan C., Bartůněk P. (2021). Will the chemical probes
please stand up?. RSC Med. Chem..

[66] Harding S. D., Armstrong J., Faccenda E., Southan C., Alexander S. P. H., Davenport A. P., Pawson A., Spedding M., Davies J., NC-IUPHAR (2022). The IUPHAR/BPS
guide to PHARMACOLOGY in 2022: curating pharmacology for COVID-19,
malaria and antibacterials. Nucleic Acids Res..

[67] Sun J., Jeliazkova N., Chupakin V., Golib-Dzib J. F., Engkvist O., Carlsson L., Wegner J., Ceulemans H., Georgiev I., Jeliazkov V., Kochev N., Ashby T. J., Chen H. (2017). ExCAPE-DB: an integrated large scale dataset facilitating Big Data
analysis in chemogenomics. J. Cheminf..

[68] Isigkeit L., Chaikuad A., Merk D. (2022). A consensus compound/bioactivity
dataset for data-driven drug design and chemogenomics. Molecules.

[69] The
UniProt Consortium (2019). UniProt:
a worldwide hub of protein knowledge. Nucleic
Acids Res..

[70] Swain, M. MolVS: Molecule Validation and Standardization, 2018.

[71] Landrum G. (2013). RDKit A software
suite for cheminformatics, computational chemistry, and predictive
modeling. Greg Landrum.

[72] Jumper J., Evans R., Pritzel A., Green T., Figurnov M., Ronneberger O., Tunyasuvunakool K., Bates R., Žídek A., Potapenko A. (2021). Highly accurate protein structure prediction
with AlphaFold. Nature.

[73] Puzyn T., Mostrag-Szlichtyng A., Gajewicz A., Skrzyński M., Worth A. P. (2011). Investigating the influence of data splitting on the
predictive ability of QSAR/QSPR models. Struct.
Chem..

[74] Song, Y. ; Zheng, S. ; Niu, Z. ; Fu, Z.-H. ; Lu, Y. ; Yang, Y. Communicative Representation Learning on Attributed Molecular Graphs. In Proceedings of the Twenty-Ninth International Joint Conference on Artificial Intelligence Main track; IJCAI, pp 2831–2838.

[75] Gu Z., Luo X., Chen J., Deng M., Lai L. (2023). Hierarchical graph
transformer with contrastive learning for protein function prediction. Bioinformatics.

[76] Jamasb A. R., Viñas R., Ma E. J., Harris C., Huang K., Hall D., Lió P., Blundell T. L. (2020). Graphein-a Python
library for geometric deep learning and network analysis on protein
structures and interaction networks. bioRxiv.

[77] Kipf T. N., Welling M. (2016). Semi-supervised classification with graph convolutional
networks. arXiv.

[78] Wang C., Zhang Y. (2017). Improving scoring-docking-screening
powers of protein-ligand scoring
functions using random forest. J. Comput. Chem..

[79] Jin Z., Wu T., Chen T., Pan D., Wang X., Xie J., Quan L., Lyu Q. (2023). CAPLA: improved prediction of protein–ligand
binding affinity by a deep learning approach based on a cross-attention
mechanism. Bioinformatics.

[80] Pei, Q. ; Gao, K. ; Wu, L. ; Zhu, J. ; Xia, Y. ; Xie, S. ; Qin, T. ; He, K. ; Liu, T.-Y. ; Yan, R. FABind: Fast and accurate protein-ligand binding. In Advances in Neural Information Processing Systems, 2024; Vol. 36

[81] Veličković P., Cucurull G., Casanova A., Romero A., Lio P., Bengio Y. (2017). Graph attention
networks. arXiv.

[82] Gilmer, J. ; Schoenholz, S. S. ; Riley, P. F. ; Vinyals, O. ; Dahl, G. E. Neural message passing for quantum chemistry. In International conference on machine learning, pp 1263–1272.

[83] Xiong Z., Wang D., Liu X., Zhong F., Wan X., Li X., Li Z., Luo X., Chen K., Jiang H. (2019). Pushing the boundaries
of molecular representation for drug discovery
with the graph attention mechanism. J. Med.
Chem..

[84] Chithrananda S., Grand G., Ramsundar B. (2020). ChemBERTa:
large-scale self-supervised
pretraining for molecular property prediction. arXiv.

[85] Wang Z., Wang S., Li Y., Guo J., Wei Y., Mu Y., Zheng L., Li W. (2024). A new paradigm
for applying deep
learning to protein–ligand interaction prediction. Briefings Bioinf..

[86] Shen T., Liu F., Wang Z., Sun J., Bu Y., Meng J., Chen W., Yao K., Mu Y., Li W. (2023). zPoseScore model for accurate and robust protein–ligand
docking
pose scoring in CASP15. Proteins: Struct., Funct.,
Bioinf..

[87] Friesner R. A., Murphy R. B., Repasky M. P., Frye L. L., Greenwood J. R., Halgren T. A., Sanschagrin P. C., Mainz D. T. (2006). Extra precision
glide: docking and scoring incorporating a model of hydrophobic enclosure
for protein-ligand complexes. J. Med. Chem..

