# Supplementary material for: Bioactivity Deep Learning for Complex Structure-Free Compound-Protein Interaction Prediction
Source: J Chem Inf Model. 2025 Sep 16;65(19):9910–26. doi: 10.1021/acs.jcim.5c00741 (PMC12529763; doi:10.1021/acs.jcim.5c00741)
Supplement: Supplementary file 1 [file ci5c00741_si_001.pdf]

SUPPORTING INFORMATION

Bioactivity deep learning for complex structure-free compound protein interaction prediction

Yaowen Gu[a], Song Xia[a], Qi Ouyang[a], Yingkai Zhang[a,b,c*]

[a]Department of Chemistry, New York University, New York, New York 10003, United States

[b]Simons Center for Computational Physical Chemistry at New York University, New York, New York 10003, United States

[c]NYU-ECNU Center for Computational Chemistry at NYU Shanghai, Shanghai 200062, China

1. Overview of benchmark dataset

The CPI2M dataset combines two sources—EquiVS and Papyrus—and we assessed their degree of overlap at multiple levels. Beyond the CPI, bioactivity, ligand, and protein overlaps presented in Figure 1, we illustrate scaffold-level redundancy (via Murcko scaffolds) in **Figure S1(A)** and activity-type overlaps in **Figure S1(B)**. These analyses confirm that EquiVS and Papyrus share a nontrivial fraction of both compounds and scaffolds. To further probe chemical and protein space coverage, we applied principal component analysis to ligand ECFP fingerprints and protein ESM-2 embeddings (**Figure S3**). The resulting distributions mirror the t-SNE visualization in **Figure 1(B)**, revealing substantial overlap between EquiVS and Papyrus in both chemical and protein feature representations.

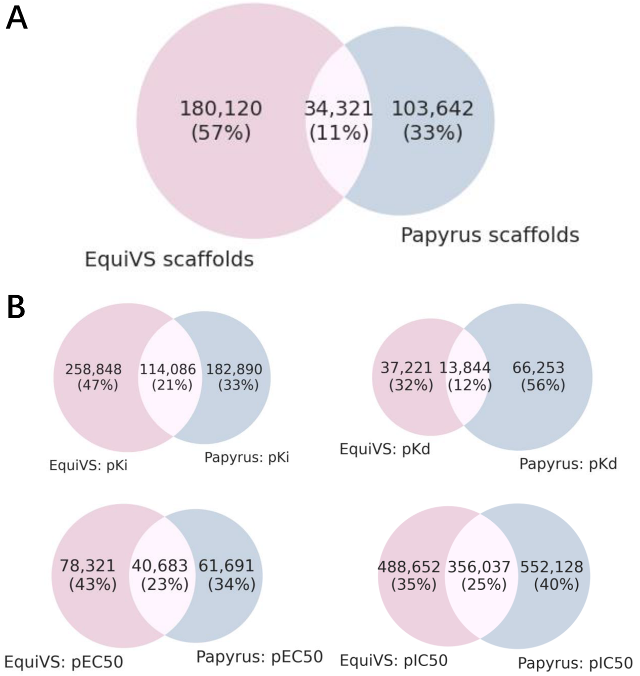

Figure S1. Overlaps in CPI2M. (A) Ligand scaffold-based overlaps; (B) Activity type-based overlaps.

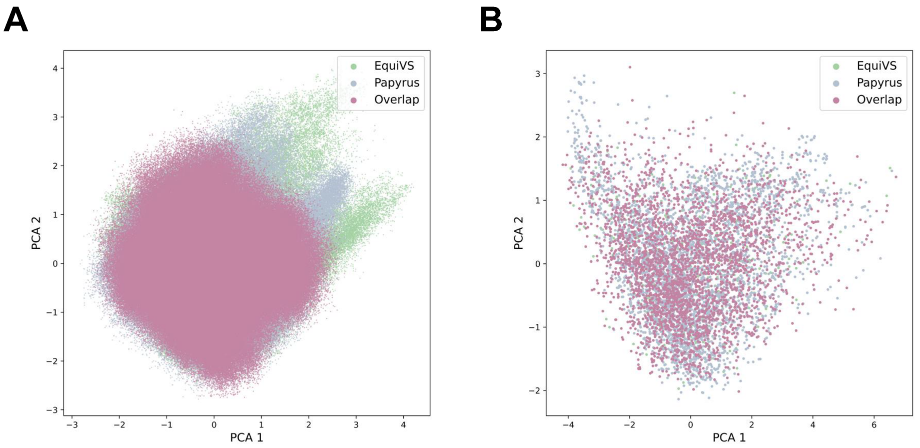

Figure S2. PCA feature deduction visualization for CPI2M. (A) Visualization for ligand chemical spaces. (B) Visualization for protein spaces.

2. Bioactivity prediction performance comparison

We assessed GGAP-CPI alongside target-specific and CPI baselines on three benchmark collections: the CPI2M-main internal validation sets, the CPI2M-few external validation sets, and the MoleculeACE transfer-learning validation sets. For general (target-independent) performance on the CPI2M-main internal sets, **Figure S3** presents Pearson's correlation coefficient (PCC) and PCC_{cliff} for GGAP-CPI and the baselines, while **Figure S4** shows Spearman's correlation coefficient (SRCC) and $SRCC_{cliff}$. For general (target-independent) performance on the CPI2M-few external sets, **Figure 5** shows activity cliff-specific performances ($RMSE_{cliff}$, PCC_{cliff}, and $SRCC_{cliff}$) for GGAP-CPI and the baseliens.

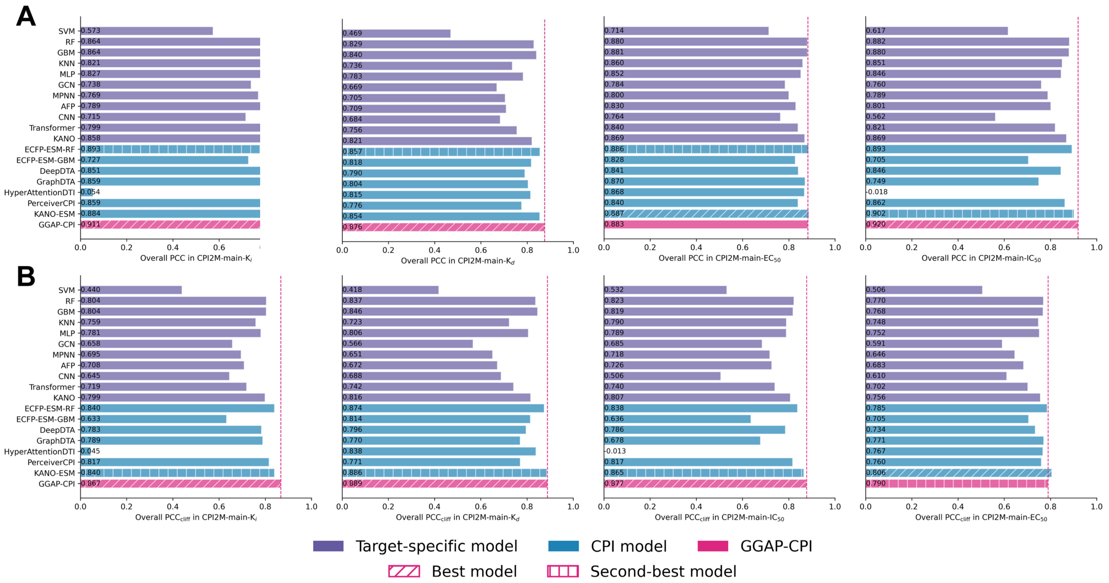

Figure S3: Comparison of overall general Pearsons' correlation coefficient (PCC) and AC-specific PCC (PCC_{cliff}) for GGAP-CPI (rose red), target-specific baselines (purple), and CPI baselines (steel blue) on the internal CPI2M-main validation sets (K_i, K_d, EC_{50}, IC_{50}). Diagonal hatching denotes the best-performing model, and vertical-line hatching denotes the second-best. Higher values indicate better performance. (A) Overall PCC; (B) Overall PCC_{cliff}.

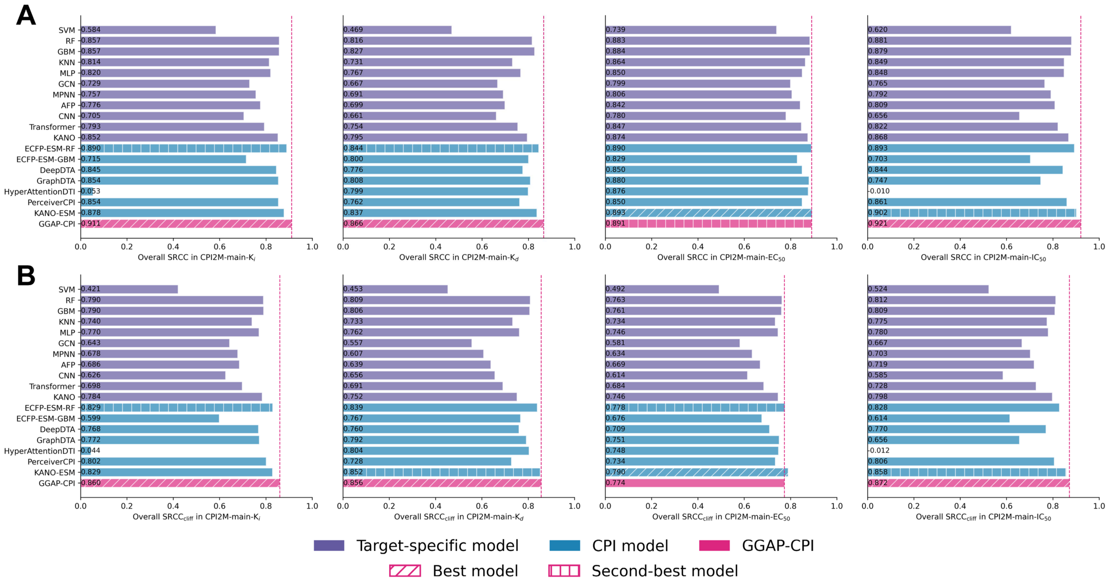

Figure S4: Comparison of overall general Spearman's correlation coefficient (SRCC) and AC-specific SRCC ($SRCC_{cliff}$) for GGAP-CPI (rose red), target-specific baselines (purple), and CPI baselines (steel blue) on the internal CPI2M-main validation sets (K_i, K_d, EC_{50}, IC_{50}). Diagonal hatching denotes the best-performing model, and vertical-line hatching denotes the second-best. Higher values indicate better performance. (A) Overall SRCC; (B) Overall $SRCC_{cliff}$.

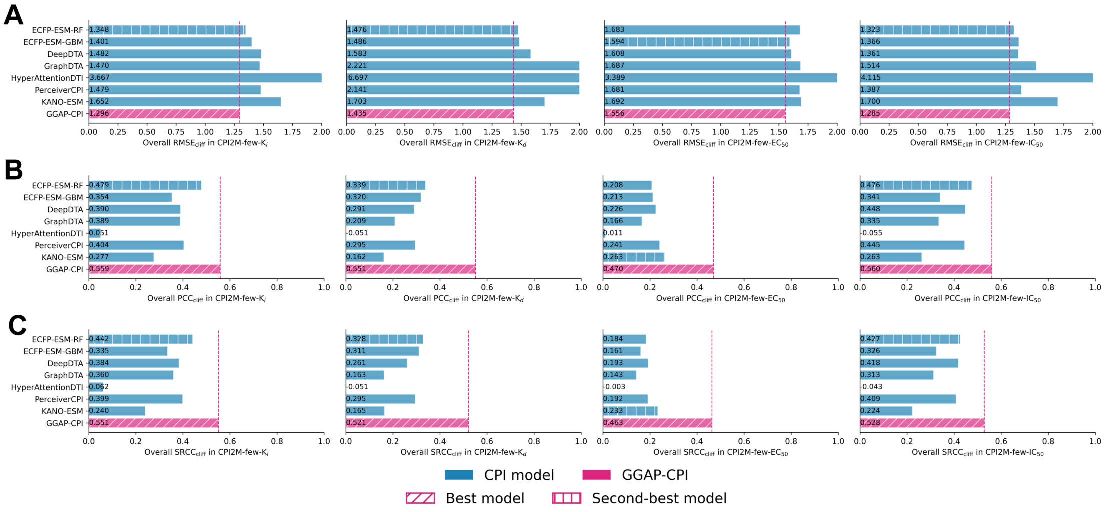

Figure S5: Comparison of overall general AC-specific performances for GGAP-CPI (rose red) and CPI baselines (steel blue) on the internal CPI2M-few validation sets (K_i, K_d, EC_{50}, IC_{50}). Diagonal hatching denotes the best-performing model, and vertical-line hatching denotes the second-best. (A) Overall $RMSE_{cliff}$; (B) Overall PCC_{cliff}; (C) Overall $SRCC_{cliff}$.

For target-specific performance, we computed metrics for each individual target and then report the mean and standard deviation across all targets. The results are organized as: **Table S1** for CPI2M-main-K_i internal validation; **Table S2** for CPI2M-main-K_d internal validation; **Table S3** for CPI2M-main-EC_{50} internal validation; **Table S4** for CPI2M-main-IC_{50} internal validation; **Table S5** for CPI2M-few-K_i external validation; **Table S6** for CPI2M-few-K_d external validation; **Table S7** for CPI2M-few-EC_{50} external validation; **Table S8** for CPI2M-few-IC_{50} external validation; **Table S9** for MoleculeACE-K_i transfer-learning validation; **Table S10** for MoleculeACE-EC_{50} transfer-learning validation. In all tables, the best-performing values are highlighted in **bold**, and the second-best values are underlined.

Table S1. Target-specific performances results of general and AC-specific metrics (RMSE, $RMSE_{cliff}$, PCC, PCC_{cliff}, SRCC, $SRCC_{cliff}$) for GGAP-CPI and baselines on the internal CPI2M-main-K_i validation sets.

Type	Model	RMSE	PCC	SRCC	$RMSE_{cliff}$	PCC_{cliff}	$SRCC_{cliff}$
Target-specific	SVM	1.140±0.383	0.575±0.141	0.566±0.139	1.331±0.387	0.499±0.301	0.486±0.302
	RF	0.718±0.231	0.750±0.144	0.707±0.165	0.874±0.252	0.673±0.279	0.641±0.280
	GBM	0.707±0.228	0.759±0.137	0.709±0.163	0.873±0.251	0.672±0.294	0.647±0.294
	KNN	0.826±0.265	0.677±0.160	0.631±0.179	0.958±0.260	0.589±0.314	0.568±0.314
	MLP	0.824±0.205	0.712±0.170	0.678±0.191	0.907±0.267	0.677±0.285	0.650±0.291
	GCN	1.059±0.287	0.546±0.215	0.497±0.226	1.193±0.336	0.490±0.363	0.464±0.361

	MPNN	1.004±0.244	0.591±0.205	0.543±0.217	1.140±0.275	0.529±0.354	0.505±0.343
	AFP	0.924±0.258	0.597±0.199	0.545±0.213	1.085±0.289	0.512±0.341	0.495±0.327
	CNN	1.039±0.355	0.550±0.179	0.513±0.185	1.176±0.327	0.476±0.346	0.461±0.340
	Transformer	0.845±0.246	0.668±0.154	0.620±0.171	1.007±0.243	0.555±0.341	0.526±0.338
	KANO	0.747±0.275	0.729±0.196	0.683±0.200	0.903±0.271	0.659±0.298	0.630±0.302
CPI	ECFP-ESM-RF	0.637±0.230	0.815±0.113	0.767±0.144	0.790±0.268	0.736±0.244	0.703±0.268
	ECFP-ESM-GBM	0.981±0.304	0.566±0.204	0.509±0.205	1.127±0.301	0.492±0.329	0.468±0.322
	DeepDTA	0.741±0.272	0.741±0.156	0.680±0.175	0.885±0.287	0.661±0.293	0.637±0.303
	GraphDTA	0.710±0.232	0.770±0.133	0.703±0.158	0.882±0.258	0.676±0.282	0.645±0.284
	HyperAttentionDTI	3.344±0.804	0.006±0.180	-0.002±0.175	3.752±0.718	0.017±0.374	0.020±0.374
	PerceiverCPI	0.720±0.287	0.750±0.163	0.700±0.176	0.843±0.315	0.705±0.283	0.678±0.293
	KANO-ESM	0.662±0.249	0.788±0.148	0.730±0.183	0.754±0.300	0.753±0.259	0.734±0.257
	GGAP-CPI	**0.571±0.262**	**0.855±0.114**	**0.805±0.140**	**0.688±0.304**	**0.811±0.208**	**0.791±0.217**

Table S2. Target-specific performances results of general and AC-specific metrics (RMSE, $RMSE_{cliff}$, PCC, PCC_{cliff}, SRCC, $SRCC_{cliff}$) for GGAP-CPI and baselines on the internal CPI2M-main-K_d validation sets.

Type	Model	RMSE	PCC	SRCC	$RMSE_{cliff}$	PCC_{cliff}	$SRCC_{cliff}$
Target-specific	SVM	1.380±0.294	0.540±0.138	0.582±0.130	1.178±0.456	0.415±0.361	0.425±0.357
	RF	0.875±0.205	0.741±0.153	0.721±0.170	0.751±0.287	0.599±0.473	0.554±0.516
	GBM	0.851±0.182	0.765±0.110	0.726±0.127	0.774±0.260	0.578±0.494	0.558±0.483
	KNN	1.069±0.259	0.650±0.141	0.653±0.123	0.953±0.311	0.580±0.262	0.547±0.335
	MLP	1.020±0.233	0.677±0.158	0.655±0.164	0.825±0.305	0.548±0.507	0.540±0.499
	GCN	1.222±0.229	0.536±0.218	0.523±0.216	1.161±0.381	0.364±0.436	0.357±0.404
	MPNN	1.214±0.195	0.563±0.219	0.523±0.219	1.120±0.372	0.385±0.490	0.328±0.455
	AFP	1.134±0.229	0.584±0.195	0.546±0.190	1.009±0.333	0.370±0.490	0.309±0.490
	CNN	1.227±0.246	0.576±0.193	0.547±0.183	1.086±0.357	0.537±0.307	0.559±0.314
	Transformer	1.026±0.193	0.657±0.169	0.651±0.151	0.926±0.269	0.456±0.443	0.447±0.412
	KANO	0.910±0.223	0.670±0.322	0.661±0.280	0.829±0.251	0.595±0.491	0.584±0.489
CPI	ECFP-ESM-RF	0.822±0.216	0.784±0.119	0.770±0.124	0.697±0.248	0.628±0.497	0.599±0.510
	ECFP-ESM-GBM	0.920±0.194	0.734±0.132	0.707±0.144	0.835±0.200	0.574±0.449	0.527±0.447
	DeepDTA	1.012±0.243	0.655±0.229	0.619±0.240	0.868±0.273	0.470±0.516	0.413±0.510
	GraphDTA	1.013±0.169	0.726±0.124	0.703±0.147	0.890±0.275	0.553±0.472	0.553±0.461
	HyperAttentionDTI	0.921±0.276	0.706±0.226	0.687±0.226	0.753±0.345	0.692±0.367	0.638±0.362
	PerceiverCPI	1.049±0.244	0.664±0.147	0.618±0.173	0.951±0.218	0.444±0.569	0.416±0.569
	KANO-ESM	0.849±0.246	0.769±0.146	0.750±0.157	0.662±0.300	0.786±0.229	**0.747±0.215**
	GGAP-CPI	**0.776±0.203**	**0.830±0.097**	**0.796±0.120**	**0.629±0.238**	**0.804±0.237**	0.736±0.330

Table S3. Target-specific performances results of general and AC-specific metrics (RMSE, $RMSE_{cliff}$, PCC, PCC_{cliff}, SRCC, $SRCC_{cliff}$) for GGAP-CPI and baselines on the internal CPI2M-main-EC_{50} validation sets.

Type	Model	RMSE	PCC	SRCC	$RMSE_{cliff}$	PCC_{cliff}	$SRCC_{cliff}$
Target-specific	SVM	1.046±0.404	0.503±0.202	0.504±0.188	1.218±0.455	0.394±0.354	0.380±0.343
	RF	0.712±0.263	0.661±0.218	0.632±0.214	0.928±0.348	0.518±0.410	0.495±0.407
	GBM	0.706±0.261	0.667±0.212	0.637±0.213	0.933±0.365	0.524±0.393	0.502±0.383

	KNN	0.784±0.288	0.591±0.233	0.565±0.236	0.948±0.313	0.504±0.355	0.472±0.352
	MLP	0.828±0.247	0.608±0.259	0.590±0.256	0.970±0.380	0.526±0.385	0.513±0.372
	GCN	1.022±0.295	0.454±0.244	0.419±0.231	1.228±0.378	0.354±0.353	0.322±0.342
	MPNN	0.983±0.264	0.512±0.243	0.473±0.245	1.170±0.376	0.386±0.402	0.349±0.400
	AFP	0.881±0.275	0.524±0.248	0.490±0.242	1.068±0.362	0.400±0.392	0.370±0.378
	CNN	0.985±0.355	0.496±0.236	0.482±0.227	1.158±0.433	0.416±0.379	0.413±0.366
	Transformer	0.824±0.284	0.582±0.215	0.546±0.222	1.041±0.323	0.426±0.387	0.399±0.372
	KANO	0.747±0.291	0.652±0.230	0.614±0.240	0.958±0.362	0.538±0.359	0.520±0.344
CPI	ECFP-ESM-RF	0.680±0.257	0.693±0.220	0.661±0.223	0.879±0.330	0.571±0.386	0.551±0.363
	ECFP-ESM-GBM	0.854±0.277	0.538±0.241	0.499±0.235	1.014±0.334	0.438±0.388	0.420±0.366
	DeepDTA	0.820±0.306	0.584±0.245	0.546±0.242	0.968±0.328	0.513±0.368	0.476±0.361
	GraphDTA	0.709±0.298	0.679±0.226	0.644±0.226	0.903±0.375	0.535±0.430	0.501±0.412
	HyperAttentionDTI	0.741±0.308	0.661±0.229	0.624±0.229	0.925±0.370	0.556±0.384	0.530±0.369
	PerceiverCPI	0.776±0.336	0.608±0.259	0.569±0.264	0.894±0.411	0.576±0.350	0.549±0.338
	KANO-ESM	**0.671±0.289**	0.703±0.230	0.668±0.239	**0.819±0.392**	0.605±0.396	0.590±0.376
	GGAP-CPI	0.695±0.361	**0.718±0.229**	**0.681±0.232**	0.850±0.422	**0.642±0.369**	**0.624±0.339**

Table S4. Target-specific performances results of general and AC-specific metrics (RMSE, $RMSE_{cliff}$, PCC, PCC_{cliff}, SRCC, $SRCC_{cliff}$) for GGAP-CPI and baselines on the internal CPI2M-main-IC_{50} validation sets.

Type	Model	RMSE	PCC	SRCC	$RMSE_{cliff}$	PCC_{cliff}	$SRCC_{cliff}$
Target-specific	SVM	1.113±0.407	0.582±0.161	0.587±0.151	1.227±0.499	0.485±0.321	0.469±0.319
	RF	0.682±0.229	0.762±0.158	0.736±0.161	0.826±0.264	0.663±0.306	0.631±0.309
	GBM	0.675±0.228	0.767±0.156	0.737±0.163	0.825±0.281	0.668±0.309	0.634±0.319
	KNN	0.763±0.247	0.703±0.177	0.677±0.177	0.882±0.268	0.612±0.312	0.585±0.310
	MLP	0.797±0.230	0.714±0.196	0.692±0.194	0.875±0.317	0.660±0.303	0.633±0.306
	GCN	1.034±0.290	0.562±0.214	0.534±0.214	1.141±0.310	0.452±0.371	0.421±0.375
	MPNN	0.990±0.263	0.604±0.217	0.574±0.214	1.089±0.298	0.509±0.340	0.472±0.349
	AFP	0.911±0.328	0.616±0.212	0.582±0.211	1.015±0.356	0.512±0.365	0.475±0.365
	CNN	1.251±0.967	0.499±0.264	0.478±0.256	1.358±1.031	0.426±0.375	0.403±0.367
	Transformer	0.829±0.251	0.686±0.168	0.656±0.173	0.982±0.289	0.571±0.311	0.534±0.313
	KANO	0.724±0.261	0.733±0.201	0.705±0.194	0.874±0.333	0.636±0.330	0.606±0.331
CPI	ECFP-ESM-RF	0.651±0.208	0.779±0.164	0.755±0.173	0.779±0.264	0.686±0.311	0.656±0.323
	ECFP-ESM-GBM	1.035±0.294	0.459±0.253	0.427±0.252	1.111±0.349	0.391±0.390	0.358±0.387
	DeepDTA	0.761±0.231	0.665±0.233	0.632±0.236	0.878±0.269	0.592±0.350	0.558±0.346
	GraphDTA	0.928±0.264	0.554±0.221	0.526±0.217	1.043±0.310	0.482±0.349	0.450±0.341
	HyperAttentionDTI	3.759±0.867	-0.010±0.190	-0.001±0.190	4.076±0.851	0.003±0.364	0.011±0.365
	PerceiverCPI	0.719±0.256	0.700±0.229	0.675±0.231	0.807±0.306	0.663±0.320	0.632±0.322
	KANO-ESM	0.628±0.232	0.776±0.191	0.750±0.203	0.708±0.309	0.723±0.319	0.693±0.331
	GGAP-CPI	**0.557±0.228**	**0.826±0.162**	**0.800±0.175**	**0.677±0.311**	**0.757±0.296**	**0.729±0.298**

Table S5. Target-specific performances results of general and AC-specific metrics (RMSE, $RMSE_{cliff}$, PCC, PCC_{cliff}, SRCC, $SRCC_{cliff}$) for GGAP-CPI and baselines on the CPI2M-few-K_i validation sets.

Model	RMSE	PCC	SRCC	$RMSE_{cliff}$	PCC_{cliff}	$SRCC_{cliff}$
ECFP-ESM-RF	1.365±0.733	0.246±0.552	0.221±0.532	1.300±0.651	0.175±0.644	0.164±0.641

ECFP-ESM-GBM	1.424±0.621	0.173±0.531	0.145±0.501	1.350±0.557	0.048±0.607	0.048±0.604
DeepDTA	1.464±0.696	0.234±0.532	0.202±0.508	1.402±0.674	0.147±0.632	0.138±0.625
GraphDTA	1.359±0.642	0.191±0.549	0.163±0.524	1.366±0.645	0.115±0.634	0.112±0.628
HyperAttentionDTI	3.099±1.157	0.012±0.479	0.008±0.475	3.372±1.099	-0.015±0.537	-0.021±0.540
PerceiverCPI	1.421±0.694	0.252±0.544	0.224±0.519	1.396±0.674	0.201±0.636	0.178±0.630
KANO-ESM	1.575±0.675	0.116±0.505	0.091±0.488	1.569±0.720	0.038±0.623	0.037±0.620
GGAP-CPI	**1.208±0.690**	**0.389±0.545**	**0.356±0.522**	**1.219±0.718**	**0.363±0.614**	**0.349±0.609**

Table S6. Target-specific performances results of general and AC-specific metrics (RMSE, $RMSE_{cliff}$, PCC, PCC_{cliff}, SRCC, $SRCC_{cliff}$) for GGAP-CPI and baselines on the CPI2M-few-K_d validation sets.

Model	RMSE	PCC	SRCC	$RMSE_{cliff}$	PCC_{cliff}	$SRCC_{cliff}$
ECFP-ESM-RF	**1.393±0.663**	0.121±0.499	0.106±0.487	**1.363±0.688**	0.174±0.702	0.164±0.702
ECFP-ESM-GBM	1.459±0.620	0.073±0.482	0.053±0.473	1.395±0.677	0.240±0.680	0.216±0.688
DeepDTA	1.560±0.689	0.124±0.498	0.106±0.503	1.480±0.726	0.164±0.703	0.157±0.709
GraphDTA	1.661±0.943	0.086±0.504	0.070±0.500	1.909±0.940	0.039±0.737	0.033±0.729
HyperAttentionDTI	5.728±1.270	-0.007±0.472	-0.004±0.467	6.236±1.333	-0.009±0.689	-0.012±0.689
PerceiverCPI	2.333±1.030	0.098±0.512	0.080±0.497	2.060±1.207	0.227±0.695	0.211±0.687
KANO-ESM	1.549±0.640	0.108±0.469	0.095±0.476	1.535±0.746	0.103±0.685	0.092±0.681
GGAP-CPI	1.448±0.631	**0.305±0.516**	**0.254±0.509**	1.399±0.606	**0.257±0.703**	**0.235±0.708**

Table S7. Target-specific performances results of general and AC-specific metrics (RMSE, $RMSE_{cliff}$, PCC, PCC_{cliff}, SRCC, $SRCC_{cliff}$) for GGAP-CPI and baselines on the CPI2M-few-EC_{50} validation sets.

Model	RMSE	PCC	SRCC	$RMSE_{cliff}$	PCC_{cliff}	$SRCC_{cliff}$
ECFP-ESM-RF	1.327±0.750	0.111±0.534	0.092±0.516	1.501±0.799	0.084±0.592	0.084±0.580
ECFP-ESM-GBM	1.242±0.678	0.097±0.524	0.085±0.505	1.421±0.695	0.076±0.571	0.071±0.568
DeepDTA	1.394±0.618	0.140±0.539	0.128±0.536	1.445±0.704	0.148±0.559	0.150±0.552
GraphDTA	1.287±0.707	0.117±0.554	0.115±0.537	1.496±0.752	0.081±0.582	0.073±0.574
HyperAttentionDTI	2.707±1.138	0.077±0.561	0.068±0.549	3.101±1.147	0.090±0.573	0.093±0.555
PerceiverCPI	1.350±0.668	0.126±0.565	0.111±0.543	1.474±0.711	0.123±0.580	0.124±0.575
KANO-ESM	1.334±0.775	0.146±0.565	0.129±0.554	1.480±0.777	0.129±0.584	0.118±0.579
GGAP-CPI	**1.195±0.678**	**0.317±0.554**	**0.289±0.537**	**1.394±0.727**	**0.300±0.572**	**0.291±0.569**

Table S8. Target-specific performances results of general and AC-specific metrics (RMSE, $RMSE_{cliff}$, PCC, PCC_{cliff}, SRCC, $SRCC_{cliff}$) for GGAP-CPI and baselines on the CPI2M-few-IC_{50} validation sets.

Model	RMSE	PCC	SRCC	$RMSE_{cliff}$	PCC_{cliff}	$SRCC_{cliff}$
ECFP-ESM-RF	1.121±0.545	0.234±0.481	0.218±0.464	1.205±0.580	0.227±0.583	0.223±0.572
ECFP-ESM-GBM	1.211±0.492	0.128±0.455	0.116±0.439	1.253±0.516	0.102±0.541	0.101±0.536
DeepDTA	1.189±0.524	0.239±0.441	0.219±0.428	1.263±0.543	0.177±0.553	0.169±0.541
GraphDTA	1.240±0.580	0.161±0.449	0.152±0.435	1.357±0.618	0.117±0.567	0.104±0.557
HyperAttentionDTI	3.511±1.005	-0.005±0.399	-0.006±0.400	3.804±1.021	-0.006±0.471	-0.003±0.469
PerceiverCPI	1.193±0.585	0.258±0.470	0.241±0.450	1.255±0.592	0.249±0.567	0.239±0.562
KANO-ESM	1.439±0.641	0.120±0.412	0.112±0.408	1.527±0.691	0.117±0.540	0.114±0.540
GGAP-CPI	**1.064±0.557**	**0.347±0.468**	**0.318±0.453**	**1.151±0.584**	**0.320±0.572**	**0.304±0.565**

Table S9. Target-specific performances results of general and AC-specific metrics (RMSE, $RMSE_{cliff}$, PCC, PCC_{cliff}, SRCC, $SRCC_{cliff}$) for GGAP-CPI-w/o ptr (trained on MoleculeACE), GGAP-CPI (trained

on CPI2M-main-train), GGAP-CPI-ft (pretrained on CPI2M-main and finetuned on MoleculeACE), and baselines (trained on MoleculeACE) on the MoleculeACE-K_i validation sets.

Type	Model	RMSE	PCC	SRCC	$RMSE_{cliff}$	PCC_{cliff}	$SRCC_{cliff}$
	SVM	0.676±0.088	0.823±0.076	0.814±0.078	0.750±0.090	0.770±0.094	0.742±0.117
	RF	0.706±0.094	0.807±0.082	0.797±0.085	0.792±0.092	0.737±0.113	0.707±0.140
	GBM	0.703±0.096	0.806±0.089	0.798±0.092	0.782±0.097	0.745±0.107	0.709±0.133
	KNN	0.742±0.101	0.787±0.091	0.779±0.093	0.842±0.098	0.700±0.112	0.659±0.152
	MLP	0.717±0.092	0.801±0.091	0.793±0.096	0.782±0.076	0.746±0.105	0.709±0.135
Target-specific	GCN	0.925±0.137	0.660±0.111	0.634±0.109	0.984±0.151	0.595±0.122	0.559±0.120
	MPNN	0.863±0.124	0.717±0.093	0.695±0.092	0.946±0.141	0.637±0.166	0.599±0.186
	AFP	0.878±0.118	0.692±0.153	0.671±0.157	0.956±0.134	0.630±0.194	0.593±0.206
	CNN	0.925±0.128	0.652±0.121	0.634±0.121	0.977±0.112	0.608±0.134	0.578±0.152
	Transformer	0.850±0.102	0.718±0.087	0.706±0.091	0.956±0.148	0.628±0.155	0.614±0.151
	KANO	0.717±0.112	0.807±0.088	0.796±0.095	0.801±0.123	0.742±0.129	0.717±0.147
	ECFP-ESM-RF	0.696±0.088	0.816±0.072	0.810±0.070	0.798±0.126	0.729±0.147	0.702±0.164
	ECFP-ESM-GBM	0.871±0.106	0.711±0.102	0.695±0.098	0.930±0.082	0.631±0.129	0.604±0.126
	DeepDTA	0.857±0.127	0.704±0.134	0.686±0.130	0.924±0.120	0.642±0.148	0.608±0.147
	GraphDTA	0.800±0.064	0.780±0.084	0.763±0.088	0.861±0.122	0.723±0.133	0.692±0.142
CPI	HyperAttentionDTI	0.749±0.098	0.781±0.082	0.762±0.088	0.827±0.110	0.715±0.123	0.683±0.142
	PerceiverCPI	0.940±0.154	0.662±0.169	0.641±0.167	0.998±0.165	0.618±0.176	0.589±0.179
	KANO-ESM	0.691±0.098	0.818±0.074	0.808±0.076	0.775±0.124	0.759±0.121	0.729±0.134
	GGAP-CPI-w/o ptr	0.686±0.093	0.820±0.072	0.807±0.080	0.779±0.122	0.751±0.125	0.728±0.127
	GGAP-CPI	0.613±0.159	**0.887±0.046**	**0.880±0.043**	**0.677±0.187**	**0.854±0.068**	**0.841±0.066**
	GGAP-CPI-ft	**0.604±0.089**	0.864±0.054	0.856±0.054	0.713±0.120	0.794±0.105	0.777±0.109

Table S10. Target-specific performances results of general and AC-specific metrics (RMSE, $RMSE_{cliff}$, PCC, PCC_{cliff}, SRCC, $SRCC_{cliff}$) for GGAP-CPI-w/o ptr (trained on MoleculeACE), GGAP-CPI (trained on CPI2M-main-train), GGAP-CPI-ft (pretrained on CPI2M-main and finetuned on MoleculeACE), and baselines (trained on MoleculeACE) on the MoleculeACE-EC_{50} validation sets.

Type	Model	RMSE	PCC	SRCC	$RMSE_{cliff}$	PCC_{cliff}	$SRCC_{cliff}$
	SVM	0.688±0.075	0.761±0.038	0.759±0.042	0.739±0.043	0.678±0.075	0.652±0.100
	RF	0.691±0.070	0.755±0.037	0.746±0.047	0.766±0.055	0.648±0.051	0.617±0.065
	GBM	0.703±0.077	0.746±0.040	0.743±0.046	0.774±0.039	0.638±0.070	0.610±0.106
	KNN	0.729±0.071	0.729±0.044	0.719±0.055	0.812±0.042	0.600±0.080	0.581±0.097
	MLP	0.738±0.094	0.723±0.056	0.718±0.057	0.791±0.068	0.638±0.084	0.605±0.099
Target-specific	GCN	0.871±0.048	0.575±0.075	0.565±0.090	0.894±0.067	0.492±0.093	0.424±0.138
	MPNN	0.820±0.057	0.651±0.047	0.636±0.059	0.865±0.124	0.544±0.043	0.476±0.132
	AFP	0.825±0.079	0.648±0.069	0.661±0.061	0.845±0.090	0.586±0.086	0.537±0.132
	CNN	0.861±0.095	0.592±0.066	0.597±0.071	0.906±0.093	0.493±0.042	0.456±0.063
	Transformer	0.804±0.066	0.671±0.044	0.673±0.053	0.870±0.085	0.561±0.069	0.563±0.070
	KANO	0.693±0.041	0.759±0.043	0.749±0.043	0.744±0.083	0.670±0.066	0.644±0.079
	ECFP-ESM-RF	0.659±0.030	0.771±0.065	0.763±0.073	0.738±0.042	0.645±0.117	0.623±0.110
CPI	ECFP-ESM-GBM	0.740±0.031	0.713±0.076	0.703±0.091	0.812±0.059	0.560±0.125	0.532±0.130
	DeepDTA	0.858±0.082	0.596±0.051	0.589±0.048	0.900±0.115	0.491±0.070	0.455±0.083
	GraphDTA	0.810±0.062	0.723±0.073	0.703±0.080	0.917±0.067	0.613±0.127	0.605±0.116

HyperAttentionDTI	0.714±0.031	0.727±0.068	0.713±0.072	0.775±0.051	0.621±0.106	0.597±0.101
PerceiverCPI	0.828±0.073	0.630±0.080	0.619±0.089	0.898±0.104	0.515±0.078	0.496±0.100
KANO-ESM	0.750±0.077	0.738±0.076	0.733±0.080	0.815±0.061	0.640±0.118	0.624±0.104
GGAP-CPI-w/o ptr	0.680±0.064	0.776±0.061	0.771±0.066	0.755±0.187	0.682±0.100	0.673±0.095
GGAP-CPI	0.742±0.150	0.784±0.100	0.794±0.094	0.760±0.147	**0.763±0.119**	**0.756±0.117**
GGAP-CPI-ft	**0.588±0.034**	**0.819±0.058**	**0.817±0.053**	**0.665±0.033**	0.718±0.104	0.706±0.088

We further evaluated GGAP-CPI on two biologically meaningful subgroups: protein families and ligand molecular-weight intervals. For Protein family subgroups (**Table S11**), we assessed the top five most populated families in the CPI2M-main internal validation set—Protein kinase superfamily, G-protein coupled receptor 1 family, ligand-gated ion channel superfamily, nuclear hormone receptor family, and Peptidase S1 family. For Molecular-weight intervals (**Table S12**): We partitioned compounds into three bins based on their molecular weights (<200 Da, 200–600 Da, >600 Da) and measured performance within each.

GGAP-CPI's accuracy varied across both dimensions. It achieved its highest predictive power on the Protein kinase superfamily—which also comprises the largest number of targets in both training and validation—and on compounds in the 200–600 Da range. These patterns indicate that GGAP-CPI's subgroup performance scales with available data volume: larger, better-represented subgroups yield more reliable predictions.

Table S11. Overall performances results of general and AC-specific metrics (RMSE, $RMSE_{cliff}$, PCC, PCC_{cliff}, SRCC, $SRCC_{cliff}$) for GGAP-CPI on data from the Top5 protein families in the CPI2M-main internal validation sets.

Dataset	Protein Family	Num. of data	RMSE	PCC	SRCC	$RMSE_{cliff}$	PCC_{cliff}	$SRCC_{cliff}$
	Protein kinase superfamily	19,833	0.409	0.926	0.889	0.719	0.892	0.884
	G-protein coupled receptor 1 family	24,696	0.669	0.877	0.877	0.751	0.834	0.835
Ki	Ligand-gated ion channel superfamily	4,843	0.555	0.934	0.939	0.536	0.926	0.919
	Nuclear hormone receptor family	612	0.943	0.838	0.792	1.144	0.770	0.730
	Peptidase S1 family	2,285	0.695	0.916	0.911	0.832	0.880	0.869
	Protein kinase superfamily	173	0.867	0.823	0.811	0.712	0.829	0.744
	G-protein coupled receptor 1 family	440	0.706	0.879	0.872	0.743	0.884	0.861
Kd	Ligand-gated ion channel superfamily	0	-	-	-	-	-	-
	Nuclear hormone receptor family	53	0.794	0.828	0.797	0.846	0.759	0.720
	Peptidase S1 family	0	-	-	-	-	-	-
	Protein kinase superfamily	615	0.740	0.822	0.851	0.885	0.722	0.742
	G-protein coupled receptor 1 family	6,239	0.784	0.859	0.858	0.877	0.789	0.775
EC50	Ligand-gated ion channel superfamily	392	1.112	0.784	0.757	1.321	0.786	0.634
	Nuclear hormone receptor family	2,455	0.769	0.841	0.840	0.938	0.685	0.667
	Peptidase S1 family	38	0.481	-0.004	-0.028	-	-	-
	Protein kinase superfamily	36,560	0.565	0.917	0.917	0.652	0.871	0.870
	G-protein coupled receptor 1 family	14,211	0.701	0.869	0.872	0.815	0.806	0.803
IC50	Ligand-gated ion channel superfamily	4,668	0.539	0.945	0.955	0.469	0.963	0.959
	Nuclear hormone receptor family	3,264	0.736	0.840	0.840	0.905	0.713	0.677
	Peptidase S1 family	2,088	0.695	0.920	0.911	0.790	0.882	0.879

Table S12. Overall performances results of general and AC-specific metrics (RMSE, $RMSE_{cliff}$, PCC, PCC_{cliff}, SRCC, $SRCC_{cliff}$) for GGAP-CPI on data from different molecular weight intervals in the CPI2M-main interval validation sets.

Dataset	Molecular Weight Interval	Num. of data	RMSE	PCC	SRCC	$RMSE_{cliff}$	PCC_{cliff}	$SRCC_{cliff}$
Ki	(0, 200]	1,405	0.731	0.881	0.874	0.873	0.862	0.854
	(200, 600]	64,592	0.583	0.910	0.910	0.716	0.862	0.857
	(600,]	3,162	0.725	0.899	0.895	0.812	0.874	0.867
Kd	(0, 200]	14	1.351	0.795	0.798	1.203	0.816	0.900
	(200, 600]	810	0.762	0.875	0.871	0.709	0.870	0.847
	(600,]	86	0.915	0.867	0.831	0.889	0.941	0.896
EC50	(0, 200]	279	0.922	0.796	0.793	0.880	0.860	0.816
	(200, 600]	16,727	0.731	0.881	0.890	0.901	0.778	0.767
	(600,]	1,002	0.790	0.864	0.846	0.937	0.828	0.811
IC50	(0, 200]	1,546	0.713	0.800	0.812	0.875	0.706	0.724
	(200, 600]	143,094	0.575	0.920	0.921	0.682	0.878	0.873
	(600,]	8,008	0.638	0.912	0.908	0.754	0.862	0.845

We next evaluated GGAP-CPI on the "cold-drug" (unseen ligand) subset of the CPI2M-few external validation data, in which 58.7% of ligands were unseen during training. **Figure S6** reports the general performance metrics (RMSE, PCC, and SRCC) for GGAP-CPI and the CPI baselines on this subset. The resulting trends mirror those observed across the full validation sets (**Figure 3**), with GGAP-CPI achieving the highest accuracy on almost every metric and dataset. Notably, in terms of PCC, GGAP-CPI outperformed the second-best baseline by 43.44%, 24.17%, 86.30%, and 14.77% on K_i, K_d, EC_{50}, IC_{50} data, respectively.

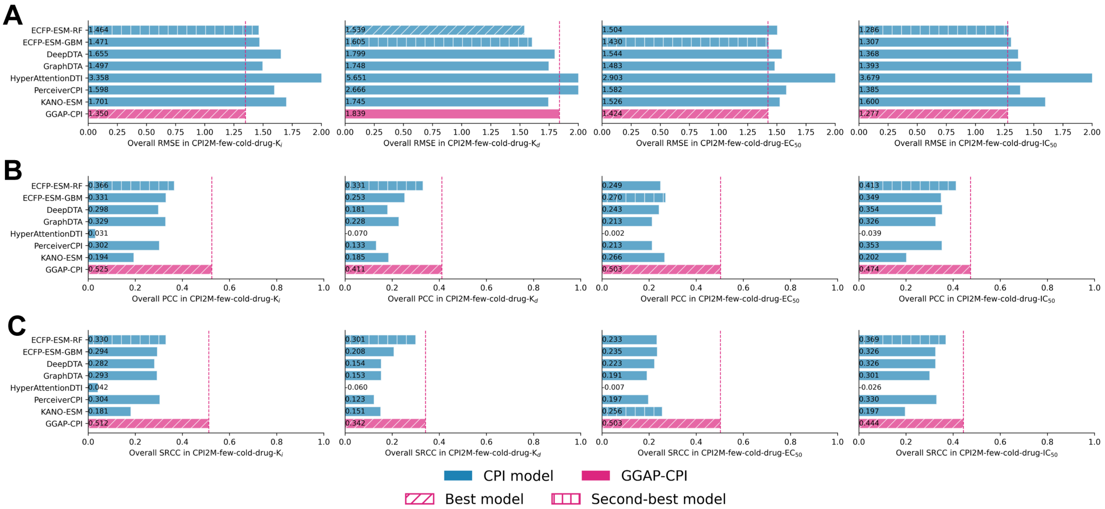

Figure S6. Comparison of overall general performance metrics—root mean squared error (RMSE), Pearson's correlation coefficient (PCC), and Spearman's rank correlation coefficient (SRCC)—for GGAP-CPI (rose red) and CPI baselines (steel blue) on the external CPI2M-few-cold-drug validation sets (K_i, K_d, EC_{50}, IC_{50}). Diagonal hatching marks the best-performing model and vertical-line hatching marks the second-best. Lower RMSE and higher PCC/SRCC values indicate better performance. (A) Overall RMSE; (B) Overall PCC; (C) Overall SRCC.

3. Introduction of compared scoring functions

We compated our GGAP-CPI as a CPI-based sorcing function (SF) with multiple other SFs, including traditional SFs (AutoDock Vina, GlideScore-XP, DyScore, SCORCH, GenScore), Δ-learning SFs ($\Delta_{Vina}RF_{20}$, $\Delta_{Lin\_F9}XGB$), CPI-SFs (the baselines reproduced in our study, SSM-DTA, BIND), DL-docking SFs (CarsiDock, DeepDock, KarmaDock), and DL-SFs (RTMScore, KDEEP, PIGNet, PIGNet2, GenScore, GatedGCN_ft_1.0, OnionNet). A brief introduction of each of these compared SFs are listed below:

Traditional-SFs:

- AutoDock Vina: Employs a hybrid empirical scoring function combining intermolecular (van der Waals, electrostatics) and intramolecular strain terms, parameterized against PDBbind affinities to estimate binding free energies.
- GlideScore-XP: Uses an extra-precision empirical function with physics-based interaction terms, hydrophobic enclosure rewards, and strict penalties for desolvation and electrostatic violations to improve pose ranking.
- DyScore: Integrates dynamic interaction features via ensemble boosting to distinguish actives from decoys.
- SCORCH: A consensus machine-learning scoring function trained on multi-pose augmented data and property-matched decoys.
- GenScore: Utilizes gradient boosting on physics-inspired structural and interaction descriptors to predict binding affinities, combining multiple regression trees for robust generalization.

Δ-learning SFs (models trained to predict the difference between a traditional scoring function's output and the true experimental affinity, effectively learning to correct the classical score):

- $\Delta_{Vina}RF_{20}$: Enhances AutoDock Vina by appending 20 additional descriptors into a random-forest framework.
- $\Delta_{Lin\_F9}XGB$: Applies eXtreme Gradient Boosting to a set of Gaussian interaction descriptors (Lin_F9).

DL-docking (methods begin with separate protein and ligand structures and use a unified deep-learning pipeline to generate poses and score affinities):

- CarsiDock: Pre-trains on large-scale predicted complexes, uses a neural network to infer protein–ligand atomic distance matrices, then reconstructs high-accuracy poses via geometry optimization
- DeepDock: Learns a target-specific statistical potential via geometric deep learning on distance likelihoods tailored per ligand-target pair, outperforming classical scoring in docking and screening tasks.
- KarmaDock: Implements a lightweight multi-stage learning pipeline that jointly optimizes docking pose generation and scoring, achieving ultrafast (~17 ms/molecule) screening with leading accuracy

DL-SFs (deep-learning scoring functions that take a pre-existing protein–ligand complex structure as input and directly predict binding affinity):

- RTMScore: Employs a graph-transformer to model residue–atom distance likelihoods via a mixture-density network, providing both scoring and uncertainty estimation for pose ranking.

- KDEEP: Applies 3D convolutional neural networks on voxelized protein–ligand complexes to predict absolute binding affinities with high throughput and accuracy, trained on large crystallographic datasets
- PIGNet: A physics-informed deep model that predicts atom–atom interaction energies parameterized by neural networks, summing them to give binding affinities and generalizing across diverse poses.
- PIGNet2: Extends PIGNet with data-augmentation strategies and a physics-informed graph neural network.
- GatedGCN_ft_1.0: Fine-tunes a gated graph convolutional network on large CPI datasets, capturing complex topology of both ligand graphs and protein residue graphs for affinity prediction
- OnionNet: Uses a multi-layer convolutional neural network on inter-molecular contacts grouped by distance shells ("onion layers"), capturing both local and nonlocal interactions for binding affinity prediction

CPI-SFs (methods only take separate protein and ligand sequences/structures to predict binding affinities):

- SSM-DTA: Combines multi-task affinity prediction and masked-language modeling in a semi-supervised framework, leveraging unpaired molecules/proteins to overcome data scarcity in DTA prediction
- BIND: Utilizes pretrained protein embedding for compound-protein interaction prediction and trained on large CPI datasets (BindingDB) to predict affinities directly from sequence and graph representations.

4. Virtual screening assessment

To assess how protein input form (e.g., computational predicted structures, experimentally validated crystal structures) influences CPI model performance, we evaluated GGAP-CPI and several baseline methods on three virtual-screening benchmarks: CASF-2016, Merck FEP, and LIT-PCBA. For CASF-2016 and Merck FEP, scoring power was measured by Pearson's correlation coefficient and ranking power by Spearman's correlation coefficient (**Tables S11–S13**), while LIT-PCBA results appear in **Tables S14–S15**. In nearly every case, replacing crystal structures with AlphaFold2-predicted coordinates or full-length sequences yielded significantly better performance, with one exception: GGAP-CPI-ft (fine-tuned on PDBBind) on CASF-2016. We attribute this overall performance gain to the fact that using AlphaFold2-predicted structures at inference time closely matches the structural distributions and conformational modes present in our training data (which also rely on full length protein's AlphaFold2 inputs). Consequently, GGAP-CPI can robustly predict bioactivity and support large-scale virtual screening without requiring experimental crystal structures. It should be noted that the results for BIND are reproduced based on the released model[1]. Other CPI-SFs are trained on CPI2M-main-IC_{50}.

Table S13. Overall performance on the CASF-2016 dataset.

Method	Crystal structure used		Alphafold2-predicted structure used	
	Scoring Power	Ranking Power	Scoring Power	Ranking Power

ECFP-ESM-RF	0.465	0.389	0.521	0.536
ECFP-ESM-GBM	0.527	0.489	0.484	0.522
DeepDTA[2]	0.489	0.406	0.540	0.490
GraphDTA[3]	0.019	-0.031	0.507	0.498
HyperAttentionDTI[4]	0.068	0.000	-0.039	0.119
PerceiverCPI[5]	0.474	0.441	0.482	0.451
BIND-K_i[1]	0.524	0.451	0.688	0.575
BIND-K_d[1]	0.543	0.439	0.662	0.525
BIND-EC_{50}[1]	0.486	0.405	0.590	0.433
BIND-IC_{50}[1]	0.502	0.416	0.663	0.479
KANO_ESM	0.474	0.439	0.548	0.518
GGAP-CPI	0.679	0.649	0.684	0.667
GGAP-CPI-ft (PDBbind-v2020)	**0.857**	**0.818**	**0.852**	**0.767**

Table S14. Overall ranking power on the Merck FEP dataset with Alphafold2-predicted structures used for inference.

Target	ECFP-ESM-RF	ECFP-ESM-GBM	DeepDTA	GraphDTA	Hyper-AttentionDTI	Perceiver-CPI	BIND-K_i	BIND-K_d	BIND-EC_{50}	BIND-IC_{50}	KANO-ESM	GGAP-CPI
CDK8 (N=24)	0.715	0.475	0.200	-0.075	0.070	0.229	-0.496	-0.138	-0.252	-0.090	0.525	**0.725**
c-Met (N=15)	0.848	-0.014	0.669	0.413	0.640	0.488	0.784	0.622	0.637	0.757	0.425	**0.852**
Eg5 (N=24)	0.352	0.382	0.334	0.493	0.013	**0.526**	0.053	0.078	0.167	0.170	0.496	0.501
HIF-2α (N=38)	0.341	-0.054	0.225	0.367	-0.210	0.199	0.258	0.425	0.319	0.465	0.457	**0.545**
PFKFB3 (N=39)	0.197	-0.063	0.138	-0.216	-0.110	0.073	0.386	0.289	0.251	0.266	0.309	**0.509**
SHP-2 (N=26)	0.709	0.378	-0.287	**0.765**	0.327	0.589	0.149	0.512	0.478	0.565	0.688	0.601
SYK (N=16)	**0.756**	-0.486	0.588	-0.109	-0.230	0.376	0.688	0.403	-0.200	0.303	0.606	0.588
TNKS2 (N=12)	0.937	0.413	0.839	0.154	0.339	0.783	0.636	0.434	0.413	0.497	0.909	**0.965**
Average (N=194)	0.607	0.129	0.338	0.224	0.105	0.408	0.307	0.328	0.227	0.367	0.552	**0.661**
Total (N=194)	0.740	0.588	0.595	0.617	-0.128	0.590	0.354	0.272	0.539	0.652	0.713	**0.786**

Table S15. Overall ranking power on the Merck FEP dataset with crystal structures used for inference.

Target	ECFP-ESM-RF	ECFP-ESM-GBM	DeepDTA	GraphDTA	Hyper-AttentionDTI	Perceiver-CPI	BIND-K_i	BIND-K_d	BIND-EC_{50}	BIND-IC_{50}	KANO-ESM	GGAP-CPI
CDK8 (N=24)	0.066	0.217	0.288	-0.138	0.029	0.307	-0.208	-0.390	-0.186	-0.114	0.378	**0.655**
c-Met (N=15)	0.041	-0.328	0.335	-0.434	0.587	0.099	**0.723**	0.326	0.640	0.536	0.260	0.587
Eg5 (N=24)	0.411	0.413	0.461	0.434	-0.056	0.512	0.176	0.355	0.044	0.085	0.149	**0.564**
HIF-2α (N=38)	0.264	0.184	0.232	0.367	-0.149	0.251	0.340	-0.067	0.156	0.371	0.347	0.361
PFKFB3 (N=39)	0.053	0.138	0.234	0.361	-0.112	0.081	0.133	0.241	0.095	0.212	0.290	**0.495**
SHP-2 (N=26)	-0.171	0.343	-0.323	0.337	0.361	**0.640**	0.217	0.538	0.465	0.614	0.192	0.424
SYK (N=16)	0.150	-0.291	**0.553**	-0.441	-0.230	-0.003	-0.582	-0.232	-0.306	-0.521	-0.056	0.179
TNKS2 (N=12)	0.189	0.650	0.280	0.699	0.344	0.699	0.434	0.594	0.294	0.266	0.517	**0.867**
Average (N=194)	0.125	0.166	0.258	0.148	0.097	0.323	0.154	0.171	0.150	0.181	0.260	**0.517**
Total (N=194)	0.068	0.322	**0.367**	-0.163	-0.039	0.023	-0.195	-0.079	0.291	0.195	0.097	0.256

Table S16. Overall $EF_{1\%}$ on the LIT-PCBA dataset with Alphafold2-predicted structures used for inference.

Target	ECFP-ESM-RF	ECFP-ESM-GBM	DeepDTA	GraphDTA	Hyper-AttentionDTI	Perceiver-CPI	BIND-K_i	BIND-K_d	BIND-EC_{50}	BIND-IC_{50}	KANO-ESM	GGAP-CPI
ADRB2	0.000	0.000	0.000	0.000	5.883	0.000	5.884	0.000	0.000	5.884	5.884	17.651
ALDH1	0.783	1.194	1.082	0.895	0.000	0.895	1.082	1.324	1.063	1.977	1.194	1.045
VDR	2.595	0.153	1.374	0.763	0.153	0.763	0.305	0.305	0.611	0.611	1.832	3.359
ESR1_ago	7.855	0.000	7.855	0.000	7.855	0.000	0.000	7.855	0.000	0.000	7.855	7.855
ESR1_ant	4.555	2.277	3.416	5.693	1.139	2.277	2.277	0.000	4.555	2.277	3.416	3.416
GBA	2.454	0.000	1.841	0.614	0.614	3.068	3.681	1.841	0.614	8.589	1.227	1.841
IDH1	0.000	0.000	2.565	0.000	2.565	0.000	15.389	5.130	5.130	15.389	2.565	17.954
KAT2A	0.516	1.031	1.031	3.608	0.515	1.546	0.000	1.031	0.515	0.000	1.546	2.062
MAPK1	0.975	0.975	1.300	2.926	0.000	1.950	0.975	0.000	0.325	0.975	0.975	1.300
MTORC1	1.033	1.033	1.033	1.033	1.033	0.000	1.033	0.000	2.066	2.066	1.033	2.066
OPRK1	8.335	0.000	0.000	0.000	0.000	0.000	0.000	0.000	0.000	0.000	12.505	12.505
PKM2	0.733	0.366	0.916	1.465	0.183	0.916	1.099	0.550	3.480	2.381	0.733	1.832
PPARG	8.531	0.000	0.000	0.000	4.266	0.000	0.000	0.000	4.266	0.000	0.000	0.000
TP53	1.567	0.000	1.567	1.567	1.567	0.000	0.000	0.000	1.567	0.000	0.000	0.000
FEN1	0.278	0.278	2.501	6.390	0.278	1.945	2.223	0.278	0.000	1.111	1.945	2.223
Average	2.681	0.487	1.765	1.664	1.737	0.891	2.263	1.221	1.613	2.751	2.847	**5.007**

Table S17. Overall $EF_{1\%}$ on the LIT-PCBA dataset with crystal structures used for inference.

Target	ECFP-ESM-RF	ECFP-ESM-GBM	DeepDTA	GraphDTA	Hyper-AttentionDTI	Perceiver-CPI	BIND-K_i	BIND-K_d	BIND-EC_{50}	BIND-IC_{50}	KANO-ESM	GGAP-CPI
ADRB2	0.000	0.000	0.000	0.000	5.883	0.000	0.000	0.000	0.000	0.000	0.000	0.000
ALDH1	0.671	0.783	1.082	0.895	0.000	0.895	1.116	1.130	1.088	2.205	0.895	1.063
VDR	0.000	0.763	1.374	0.763	0.153	0.763	0.453	0.113	0.679	0.679	0.763	3.206
ESR1_ago	7.855	0.000	7.855	0.000	7.855	0.000	0.000	7.827	0.000	0.000	0.000	0.000
ESR1_ant	1.139	7.971	3.416	5.693	1.139	2.277	1.980	0.990	3.961	2.971	5.693	3.416
GBA	3.068	0.000	1.841	0.614	0.614	3.068	3.012	0.602	0.602	8.434	0.614	1.841
IDH1	2.565	2.565	2.565	0.000	2.565	0.000	12.824	7.694	2.565	12.824	0.000	17.954
KAT2A	0.515	1.031	1.031	3.608	0.515	1.546	0.516	0.516	0.516	0.516	3.608	2.062
MAPK1	0.650	1.625	1.300	2.926	0.000	1.950	1.299	0.325	0.325	0.650	2.926	1.625
MTORC1	0.000	1.033	1.033	1.033	1.033	0.000	0.000	1.033	1.033	2.066	1.033	3.099
OPRK1	0.000	4.168	0.000	0.000	0.000	0.000	8.335	4.167	0.000	0.000	0.000	12.505
PKM2	1.282	1.465	0.916	1.465	0.183	0.916	1.466	1.832	3.114	2.748	1.465	1.649
PPARG	0.000	4.266	0.000	0.000	4.266	0.000	0.000	0.000	7.462	3.731	0.000	0.000
TP53	0.000	0.000	1.567	1.567	1.567	0.000	0.000	0.000	0.000	1.280	1.567	0.000
FEN1	0.556	0.000	2.501	6.390	0.278	1.945	2.168	0.542	0.000	0.813	6.390	1.945
Average	1.220	1.711	1.765	1.664	1.737	0.891	2.211	1.785	1.423	2.594	1.664	**3.358**

5. Bioactivity uncertainty estimation

In addition to our analysis on the CPI2M-main set (**Figure 8A**), we evaluated standard deviation as an uncertainty metric on the more challenging CPI2M-few test set. As shown in **Figure S7**, the relationship between uncertainty and mean absolute error closely mirrors that observed in **Figure 8A**: subsets with lower predictive uncertainty attain significantly lower MAE. This consistency demonstrates that standard deviation remains a reliable indicator for identifying high-confidence predictions, even under sparse-data conditions.

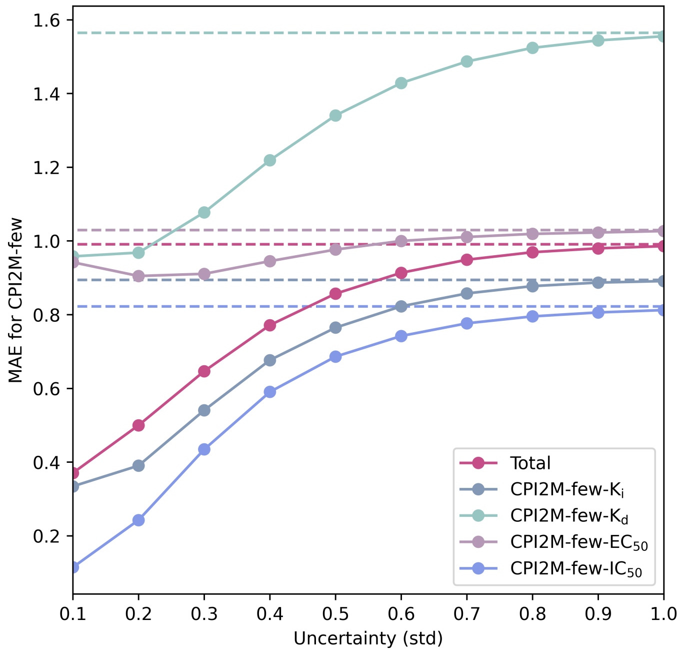

Figure S7. Subset MAE vs. uncertainty threshold for GGAP-CPI on the CPI2M-few external test sets. The corresponding uncertainty is served as the threshold to screen samples with lower uncertainty, then MAE is calculated on these subset data.

6. Data scaling effect in GGAP-CPI model training

We performed a controlled study to assess how the size of the training set affects GGAP-CPI's predictive accuracy. By training on random subsets comprising 5%, 10%, 20%, and 100% of the full dataset, we found that RMSE steadily decreased—indicating improved performance—with increasing data volume, and reached its minimum when all available training examples were used (**Figure S8**).

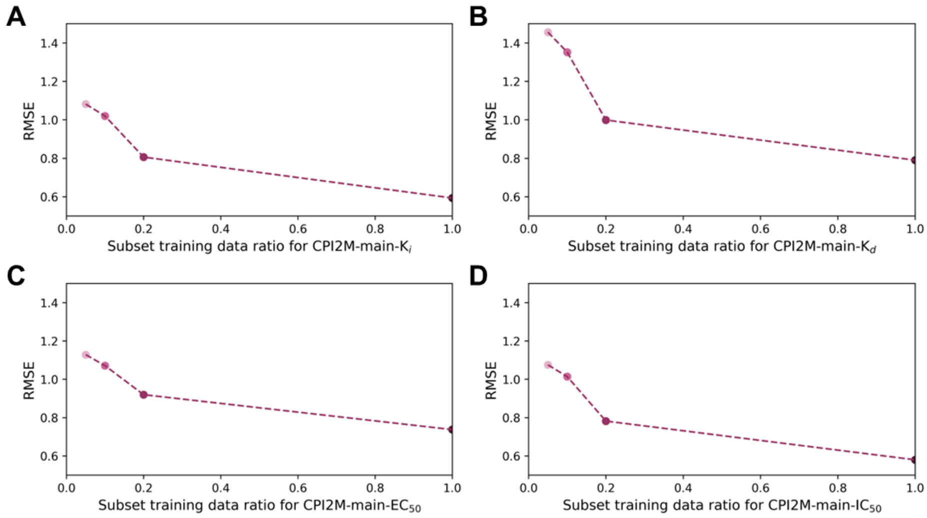

Figure S8. Overall RMSE of GGAP-CPI on the CPI2M-main internal validation sets as a function of training data fraction (5%, 10%, 20%, and 100%). Lower RMSE values denote better predictive accuracy.

7. Inference speed comparison

We compared inference speeds to assess GGAP-CPI's suitability for large-scale virtual screening. Using a generated set of one protein (UniProt ID: P42336) and 1,000 randomly sampled ligands, we measured the inference time for GGAP-CPI, its five ablation variants, and PerceiverCPI on a single NVIDIA A100 GPU with one CPU core (**Table S18**). The results show that GGAP-CPI does not introduce substantial computational overhead, remaining practical for large-scale screening. Note that the reported times of GGAP-CPI reflect a single base model; executing the full GGAP-CPI ensemble in parallel across multiple GPU cores achieves similar per-model throughput. In addition, inference performance can be further improved by employing additional CPU cores to accelerate molecular graph construction.

Table S18. Inference times for GGAP-CPI, its five variants, and PerceiverCPI on one target (P42336) with 1,000 ligands.

Model	Inference time (s)
PerceiverCPI	8.55
GGAP-CPI-w/o Mol. Encod.	16.51
GGAP-CPI-w/o Prot. Encod.	17.39
GGAP-CPI-w/o ESM-2 Emb.	21.07
GGAP-CPI-w/o CroAtt. Pool.	16.06
GGAP-CPI-w/o Int. Ensem.	16.64
GGAP-CPI	17.58

8. CPI data collection, preprocessing, and cleaning

We curated the CPI2M benchmark via a multi-step filtration pipeline applied to EquiVS and Papyrus (**Figure S9**). After each filter, we tracked the remaining CPI entries, ending with over 4 million total records. Of these, roughly 2 million—all those reporting K_i, Kd, EC_{50}, or IC_{50}—meet high-to-moderate quality standards and were used for affinity regression. Lower-quality pPotency and percentage-inhibition data were excluded from the regression models.

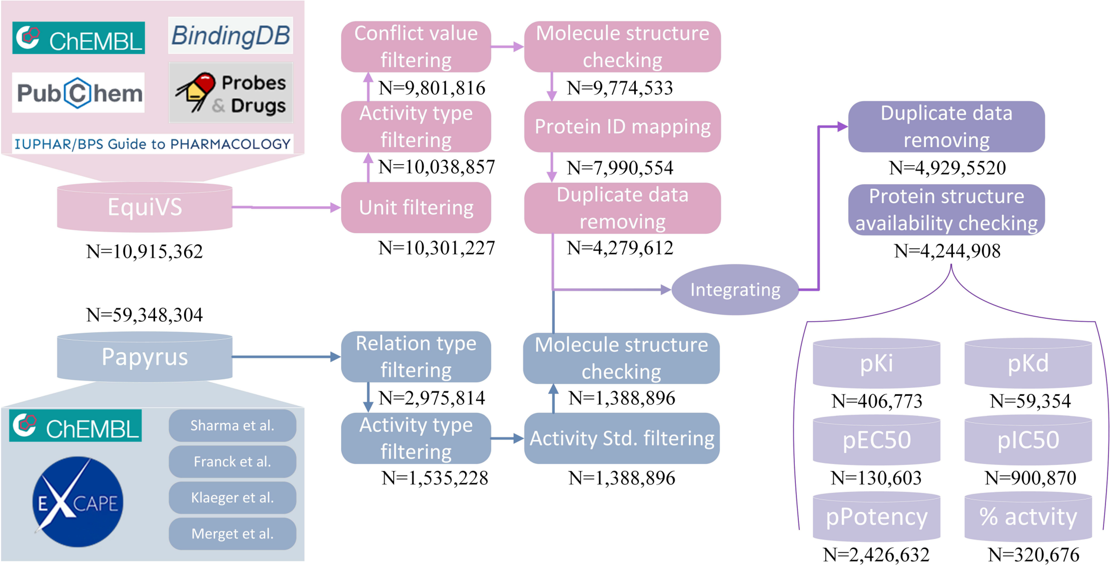

Figure S9. The data collecting, processing, and integrating workflow for CPI2M benchmark. EquiVS and Papyrus data are collected with about 70M measurements. A multi-step filtering and processing strategy is used to screen high quality CPI data from two sources. Then, the integrated CPI data after duplicate data removing and protein structure availability checking are categorized based on the activity types to generate six subsets (K_i, K_d, EC_{50}, IC_{50}, Potency, and percentage of activity) as the final candidates for CPI2M.

9. Cross attention mechanism

The calculation of cross attention mechanism in GGAP-CPI is described in **Figure S10**.

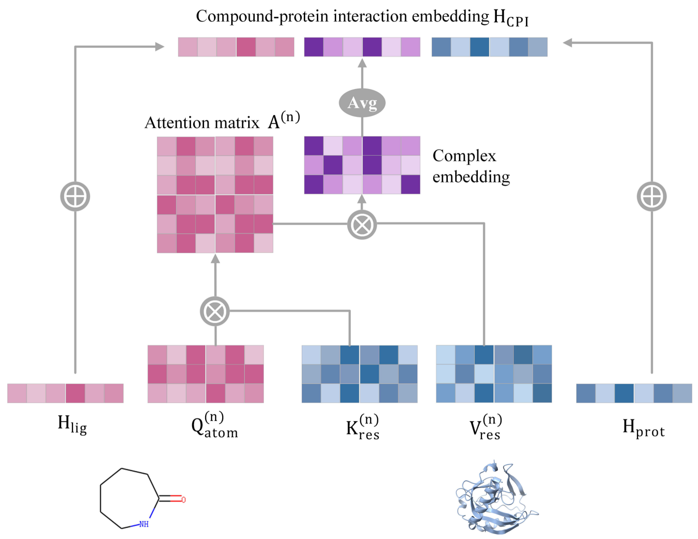

Figure S10: The computing flow in multi-head cross attention pooling module to generate CPI complex embedding from ligand and protein embeddings. The operator "⊕" represents concatenation, and "⊗" represents inner product with linear transformation and non-linear activation.

10. Integrated bioactivity learning process

We implemented an integrated bioactivity learning scheme to train GGAP-CPI, with the dual aims of improving overall performance via ensemble learning and enriching the structural feature space by pooling data from multiple activity types. As shown in **Figure S11**, we first combined all activity-type training examples into a single corpus and then performed two independent 5-fold cross-validations, yielding ten GGAP-CPI models in total.

- Global performance models (first 5): Validation folds were drawn by simple random partitioning of the merged dataset, facilitating selection of models with maximal overall accuracy across the full data distribution.
- Balanced-tail models (second 5): Validation folds were constructed via stratified random sampling of K_i measurements across discrete bioactivity intervals, producing more uniformly distributed validation sets that upweight rare or extreme samples.

The first ensemble captures GGAP-CPI's capacity for broad, high-accuracy prediction, while the second ensemble emphasizes robustness on high-quality and long-tail instances. After the model training is finished, all 10 models were ensembled with an average calculation to form the final GGAP-CPI consensus model.

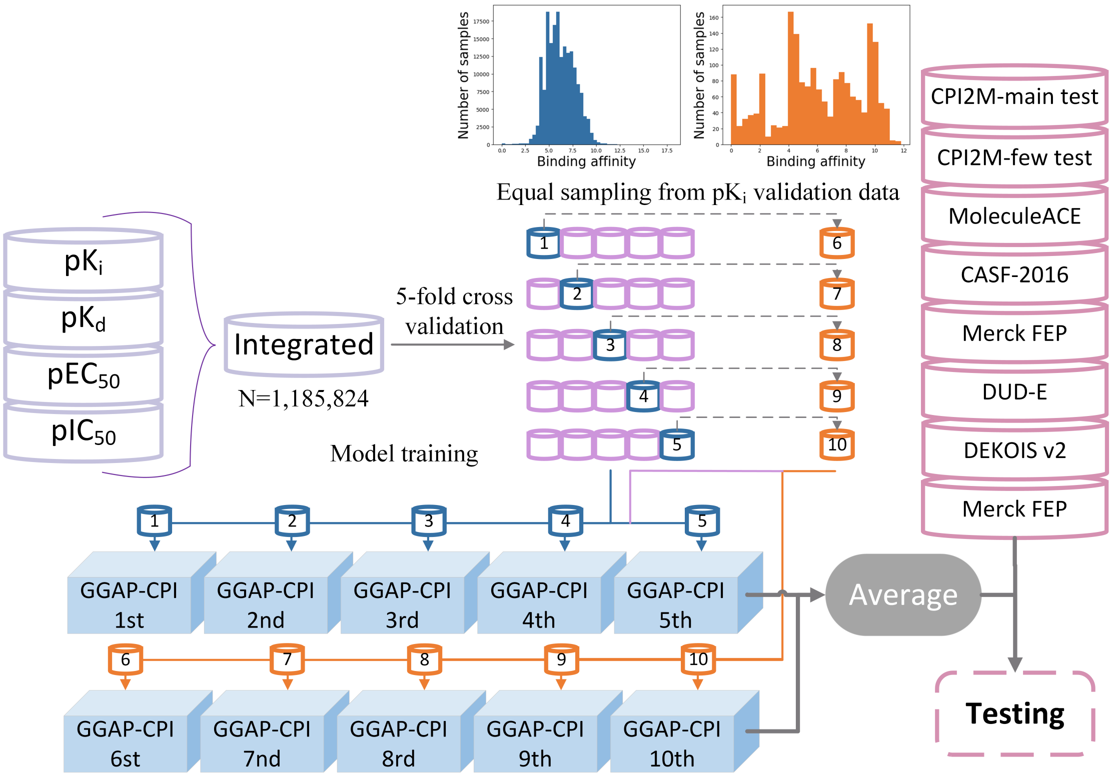

Figure S11. The integrated bioactivity learning process for GGAP-CPI.

11. Reference

(1) Lam, H. Y. I.; Guan, J. S.; Ong, X. E.; Pincket, R.; Mu, Y. Protein language models are performant in structure-free virtual screening. *bioRxiv* **2024**, 2024.2004. 2016.589765.
(2) Öztürk, H.; Özgür, A.; Ozkirimli, E. DeepDTA: deep drug–target binding affinity prediction. *Bioinformatics* **2018**, *34* (17), i821-i829.
(3) Nguyen, T.; Le, H.; Quinn, T. P.; Nguyen, T.; Le, T. D.; Venkatesh, S. GraphDTA: predicting drug–target binding affinity with graph neural networks. *Bioinformatics* **2021**, *37* (8), 1140-1147.
(4) Zhao, Q.; Zhao, H.; Zheng, K.; Wang, J. HyperAttentionDTI: improving drug–protein interaction prediction by sequence-based deep learning with attention mechanism. *Bioinformatics* **2022**, *38* (3), 655-662.
(5) Nguyen, N.-Q.; Jang, G.; Kim, H.; Kang, J. Perceiver CPI: a nested cross-attention network for compound–protein interaction prediction. *Bioinformatics* **2023**, *39* (1), btac731.
